# Role of Altered Metabolites and Metabolic Pathways in Major Tuber Crops Under Drought Stress

**DOI:** 10.1002/pei3.70126

**Published:** 2026-02-13

**Authors:** Maltase Mutanda, Fikile N. Makhubu, Sandiswa Figlan

**Affiliations:** ^1^ Department of Agriculture and Animal Health University of South Africa Florida South Africa

**Keywords:** cassava, drought tolerance, food security, metabolites, potato, sweet potato

## Abstract

Drought stress poses a significant challenge to growth and productivity of major tuber crops, particularly cassava ( *Manihot esculenta* Crantz), potato ( *Solanum tuberosum* L.) and sweet potato ( *Ipomoea batatas* (L.) Lam.). These crops are among the most widely cultivated tubers globally and play a critical role in food and nutritional security, especially in drought‐prone regions of sub‐Saharan Africa, Asia and Latin America. Several studies have highlighted that metabolites such as sucrose, proline and arginine contribute to osmotic adjustment, cellular protection and energy balance under drought stressed conditions. However, a comprehensive synthesis of drought‐induced metabolic responses and associated pathways utilized by major tuber crops remains limited. Therefore, this study aimed to identify and evaluate the metabolic responses and pathways altered under drought stress in major tuber crops (cassava, potato and sweet potato). A cross‐study analysis of peer‐reviewed research articles retrieved from Web of Science and Scopus databases identified 223 metabolites reported to be significantly altered under drought‐stressed compared with non‐stressed conditions across 30 original research articles published between 2010 and 2024. MetaboAnalyst platform was used to map metabolites to their respective pathways and to quantify pathway enrichment. The higher number of drought‐responsive metabolites was reported in potato, followed by sweet potato, reflecting the greater availability of metabolomics studies for these crops, whereas the limited metabolite information for cassava is attributable to fewer published studies rather than reduced drought responsiveness. Trehalose and proline were found to be the most commonly studied and highly affected metabolites across the three crops. Enriched metabolic pathways included glyoxylate and dicarboxylate metabolism, the citrate cycle, alanine, aspartate and glutamate metabolism, galactose metabolism, and starch and sucrose metabolism. The present findings clearly call for further research to expand metabolomics investigations, particularly in cassava which is widely promoted for its climate resilience across sub‐Saharan Africa, to unravel regulatory mechanisms linking metabolites, gene expression and drought‐adaptive phenotypes. Therefore, an integration of metabolomics with transcriptomics and proteomics could also provide a more comprehensive overview of the tuber crops' responses to drought stress, helping in accelerating breeding efforts while ensuring improved food security.

## 1 Introduction

Tuber crops are ranked third among food crops based on global production after cereal and legume crops (Chauhan et al. 2022). They form a substantial part of the world's food supply, contributing significantly to the nutritional needs of populations globally (Chandrasekara and Josheph Kumar 2016). Tuber crops, including potato ( *Solanum tuberosum* ), cassava ( *Manihot esculenta* ) and sweet potato ( *Ipomoea batatas* L.) are widely cultivated due to their ability to thrive in diverse environmental conditions, producing optimal yields even in suboptimal climates (Nanbol and Namo 2019). Despite their significance and resilience, tuber crops are still vulnerable to the adverse effects of drought stress, which can severely impact their growth and yield (Begna 2021; Makhubu et al. 2025; Mutanda, Amelework, et al. 2025). Under drought‐stressed conditions, plants develop a range of strategies of adaptations at different levels of organization including morphological, physiological, biochemical and metabolic adjustments to cope with water scarcity (Shah and Satti 2023; Khamis et al. 2025; Mutanda, Shimelis, et al. 2025). Therefore, understanding these mechanisms at the metabolic and biochemical levels is crucial for developing drought‐tolerant varieties that can maintain high productivity under limited water conditions.

Building on their inherent adaptability and resilience, recent advances in plant metabolomics have significantly expanded scientists' understanding of how tuber crops respond to drought stress conditions. Unlike genomics and transcriptomics, which mainly analyze gene expression patterns, metabolomics offers a direct snapshot of the plant's physiological state under drought‐stressed conditions (Almeida et al. 2020; Qiu et al. 2023). Metabolomics, which involves the comprehensive study of metabolites, provides valuable insights into the molecular mechanisms that drive drought response. This approach focuses on analyzing the metabolites such as sugars, amino acids, organic acids and secondary metabolites (such as phenolic acids and flavonoids). The changes in these metabolites can influence critical processes, including osmotic regulation and maintenance of cellular integrity (Mutanda, Amelework, et al. 2025; Mutanda, Shimelis, et al. 2025). However, specific directional changes (e.g., accumulation of osmolytes or antioxidants) are associated with enhanced drought tolerance (Arbona et al. 2013; Drapal et al. 2017), but the effect depends on tissue, timing and overall plant physiological. Importantly, drought‐induced shifts in metabolite levels can indicate either adaptive processes or stress‐related damage such as increased malondialdehyde (MDA) showing lipid peroxidation and membrane injury (Zhang, Yan, et al. 2021; Zhang, Luan, et al. 2021).

Tuber crops' metabolomics has proven instrumental in identifying and characterizing a diverse array of metabolites that contribute to plant health and resilience (Evers et al. 2010; Barnaby et al. 2015; Yang et al. 2015; Drapal et al. 2017; Zhou et al. 2022). The application of metabolomics in drought research has provided deeper insights into how tuber crops regulate their metabolic networks to cope with water scarcity. Furthermore, other studies have shown that tuber crops exhibit shifts in carbohydrate metabolism, alterations in organic acid and nitrogen metabolism, and the activation of pathways that help maintain energy balance during periods of water scarcity (Rosado‐Souza et al. 2019; Obata et al. 2020; Zhou et al. 2022; Yin, Chen, et al. 2024; Yin, Qiao, et al. 2024). These metabolic adjustments enhance the plant's ability to conserve water, sustain cellular functions, and maintain growth under drought‐stressed conditions.

Furthermore, drought stress triggers the production of reactive oxygen species (ROS), which can damage vital cellular components leading to oxidative stress and potential cellular dysfunction (Cruz de Carvalho 2008; Mutanda, Amelework, et al. 2025; Mutanda, Shimelis, et al. 2025). However, plants including tuber crops use antioxidant defense mechanisms to mitigate ROS‐induced damage. These defenses include the accumulation of metabolites such as sucrose, proline, ascorbate and glutathione, which help maintain cellular turgor and protect cellular membranes from dehydration under water stress (Sachdev et al. 2021). Notably, proline, a well‐known osmolyte, plays a crucial role in stabilizing proteins and cellular structures while also acting as an antioxidant to scavenge ROS (Ghosh et al. 2022). The accumulation of proline and other compatible solutes helps preserve cellular membrane integrity and sustain essential physiological functions under drought‐stressed conditions (Mutanda et al. 2024). Therefore, these metabolite‐driven defense strategies enable tuber crops to survive and maintain productivity in drought‐prone environments.

Despite these significant advances in understanding the metabolic responses to drought stress in crops, substantial gaps remain in understanding the full spectrum of metabolic changes that occur under drought‐stressed conditions. While much is known about the role of specific metabolites associated with drought tolerance, the metabolic responses and pathways in major tuber crops (such as cassava, potato and sweet potato) are largely unexplored. The metabolic responses to drought stress are highly dynamic and can vary between different crops, genotype, developmental stage, tissue type, drought severity, duration and the method of drought stress imposition (Burnett et al. 2021; Makhubu et al. 2024; Mutanda et al. 2024; Khamis et al. 2025; Makhubu et al. 2025). This variability highlights the need for a more comparative approach to studying drought tolerance of tuber crops at their metabolic level. Therefore, this study aimed to evaluate the metabolic responses and pathways under drought stress in major tuber crops (cassava, potato and sweet potato). The findings will provide valuable insights to future breeding programs by identifying the key metabolites and pathways that could serve as species‐non‐specific biomarkers to enhance drought resilience and productivity of tuber crops for sustainable agriculture.

## 2 Materials and Methods

### 2.1 Literature Search

**FIGURE 1:**
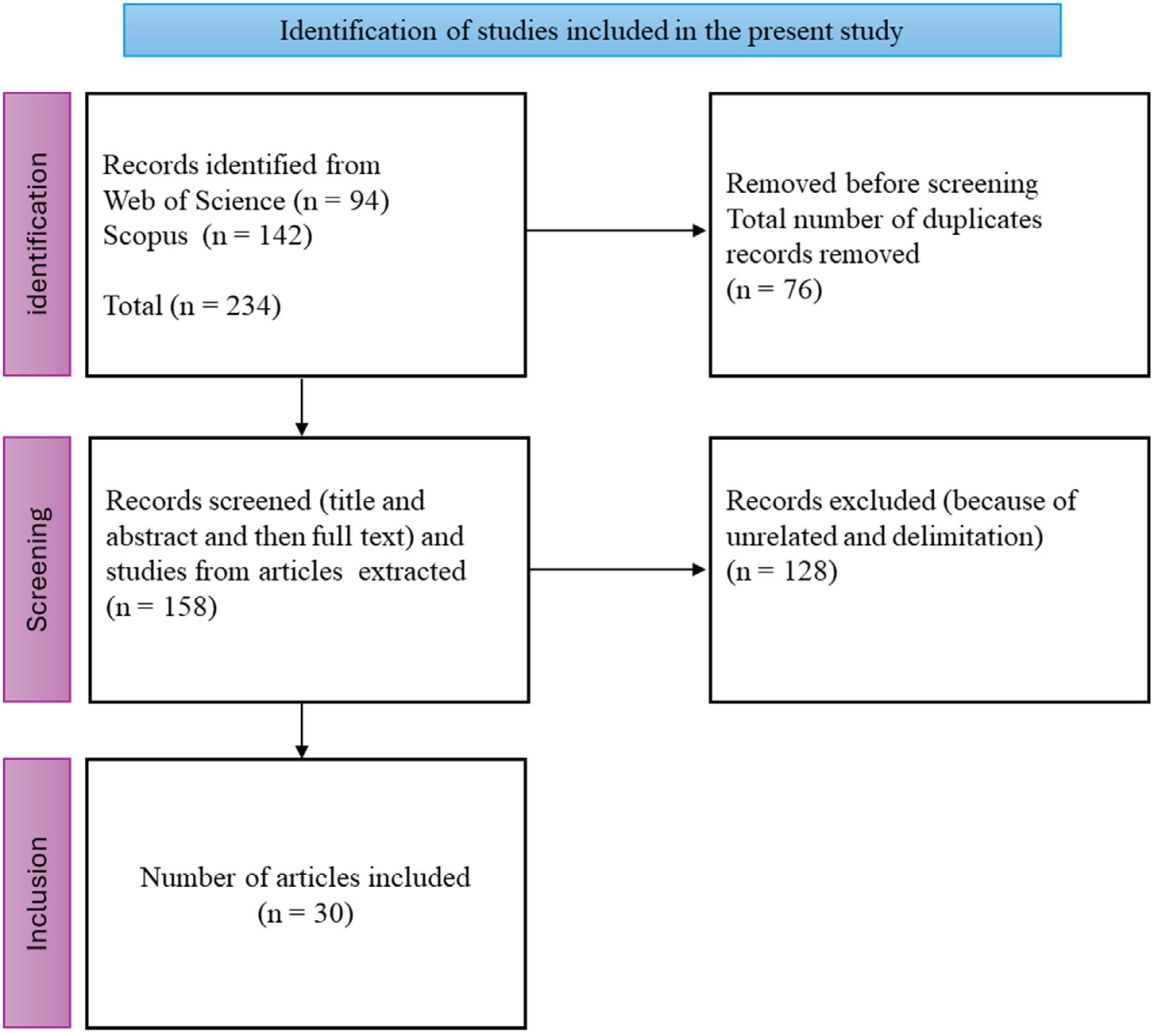
Articles selection process for the present study.

The Preferred Reporting Items for Systematic Reviews and Meta‐Analyses (PRISMA) guidelines were strictly followed in designing, conducting, and reporting this review. This systematic narrative review adhered to the PRISMA 2020 statement, ensuring transparent identification, screening, eligibility assessment and inclusion of studies. The PRISMA 2020 checklist and flow diagram (as recommended by Page et al. 2021) were used to guide the reporting, and because this review used published literature only, ethical approval was not required. A systematic search of literature was conducted from two bibliographic databases [Web of Science (https://www.webofscience.com/) and Scopus (https://www.scopus.com/)]. The search for articles from Google Scholar was avoided due to the high number of irrelevant results retrieved. The studies were reported in English by peer‐reviewed journals in the period between 2010 and 2024 (inclusive). The period from 2010 to 2024 ensures the inclusion of recent research, reflecting the latest advancements, methodologies and technologies in the field. This timeframe ensures the studies remain relevant to current scientific understanding and practices. The search on academic databases was performed on November 5, 2024. The keywords that facilitated the search of articles were “cassava”, “sweet potato”, “potato” “drought stress”, “limited water conditions”, “water stress”, “metabolite”, “metabolite profiling” and “metabolic responses”. We included peer‐reviewed research studies conducted under open‐field and controlled environmental conditions. The study selection procedure is summarized in the PRISMA flow diagram (Figure 1), and all publications included in the review as well as the metabolites altered by drought stress are listed in Table S1. The present study only included metabolites that showed statistically significant alterations under drought‐stressed compared to non‐stress conditions in major tuber crops. For pathway analysis, metabolite names reported across the selected studies were standardized by matching them to their corresponding entries in the Human Metabolome Database (HMDB, https://hmdb.ca/ accessed January 20, 2025) and the Kyoto Encyclopedia of Genes and Genomes (KEGG, https://www.genome.jp/kegg/ accessed January 20, 2025) using default database settings. The list of metabolites was then imported into MetaboAnalyst 5.0 for pathway enrichment and topology analysis. The pathway enrichment was performed using Fisher's exact test, and pathway impact was assessed using relative‐betweenness centrality, following the default parameters in MetaboAnalyst. All steps were performed using default settings.

## 3 Results

### 3.1 Literature Search

**FIGURE 2:**
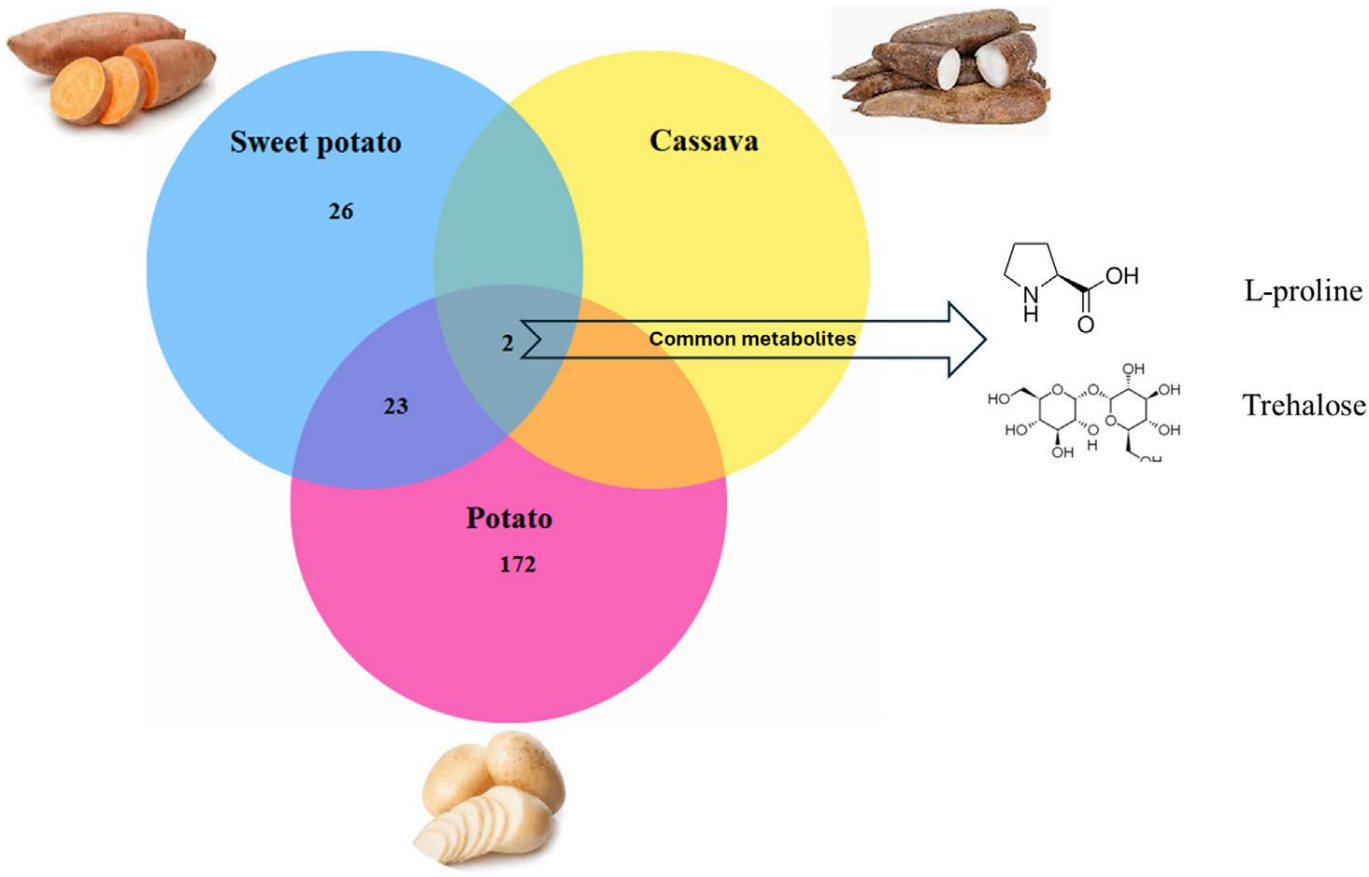
Venn diagram showing the significantly affected metabolites in three major tuber crops (sweet potato, cassava and potato) under drought‐stressed conditions. The numbers within each section represent the count of unique or shared metabolites among the three crops. Sweet potato exhibits 26 unique metabolites, cassava has no unique metabolite, and potato contains 172 distinct metabolites. Twenty‐three metabolites are shared between sweet potato and potato, while only two metabolites are common across all three crops. The common metabolites, depicted with their chemical structures, include proline and trehalose based on screened studies.

After checking the relevance according to the titles, the initial search identified a total of 234 studies published from 2010 to 2024. A detailed review of titles, abstracts and full text according to the selection criteria resulted in a total of 30 peer‐reviewed research articles reporting original experimental data (reviews, editorials, and book chapters were excluded), and all of them were reported in English from all continents. There was a drastic increase in publications from 2010 to 2024, with many of them focusing on potato drought stress responses. A total of 223 metabolites were identified as being influenced by drought stress and they were extracted from a total of 30 peer‐reviewed research articles. Of these, 25 metabolites were found in two or more crops and reported in more than three studies each. Notably, two metabolites (L‐proline and trehalose) exhibited significant changes under drought stress responses across three crops reviewed in the present study (Figure 2). Table S1 provides an overview of the selected studies and metabolites significantly altered under drought‐stressed conditions.

### 3.2 Metabolic Responses

Table S1 summarizes the presence of various metabolites identified in cassava, potato, and sweet potato, along with supporting references. A diverse range of metabolites, including sugars, amino acids, organic acids and phenolic acids were identified across the three crops. Trehalose was found to be a common sugar in all three crops. Sweet potato exhibited a unique profile with the presence of xylose, which was not detected in cassava or potato. On the other hand, raffinose was identified in potato plants only. Potato exhibited the most extensive metabolite profile, with a significantly larger number of unique metabolites detected compared to cassava and sweet potato. Sweet potato showed a distinct metabolite composition, characterized by the presence of several compounds not found in cassava or potato, highlighting specialized metabolic pathways.

### 3.3 Pathway Analysis

The present analysis revealed the involvement of multiple metabolic pathways associated with drought stress adaptation in tuber crops which are highlighted in Table 1 and Figure S1. Glyoxylate and dicarboxylate metabolism (GDM) was highly enriched, with key metabolites including malate, oxaloacetate, citrate and succinate, indicating its role in carbon assimilation and energy conservation. The citrate cycle (TCA cycle) was also significantly represented, with malate, succinate, oxaloacetate, fumarate and citrate detected. The identification of phosphoenolpyruvate and pyruvate suggests an active link between glycolysis and the TCA cycle, supporting carbon flux regulation. The alanine, aspartate, and glutamate metabolism pathway showed enrichment with aspartate, alanine, oxaloacetate, asparagine and 2‐oxo‐glutarate. The carbohydrate metabolism pathways were prominently represented, with galactose metabolism showing the accumulation of stachyose, raffinose, sucrose, glucose and myo‐inositol; as well as starch and sucrose metabolism enriched with glucose, fructose, maltose, starch and trehalose.

**TABLE 1:** Significant metabolic pathways (with an impact value > 0.1 and *p* value < 0.05) detected in major tuber crops evaluated in the present study.

Pathway name	Assigned metabolites	*p*	−log(*p*)	Holm *p*	FDR	Impact
Glyoxylate and dicarboxylate metabolism	Malate, oxaloacetate, cis‐aconitate, isocitrate, citrate, glutamate, serine, succinate, glycine, 2‐axo‐glutarate, glycerate	1.37 × 10^−8^	7.86	1.25 × 10^−6^	1.20 × 10^−6^	0.48
Citrate cycle (TCA cycle)	Malate, succinate, oxaloacetate, fumarate, citrate, Phosphoenolpyruvate, cis‐aconitate, iso‐citrate, 2‐axo‐glutarate, pyruvate	2.64 × 10^−8^	7.58	2.37 × 10^−6^	1.20 × 10^−6^	0.45
Alanine, aspartate, and glutamate metabolism	Aspartate, Alanine, oxaloacetate, asparagine, pyruvate, 2‐axo‐glutarate, fumarate, glutamate, 4‐aminobutanoate, succinate	8.39 × 10^−8^	7.08	7.47 × 10^−6^	2.55 × 10^−6^	0.70
Galactose metabolism	Stachyose, raffinose, myo‐Inositol, melibiose, sucrose, glucose, fructose, glycerol, mannose, Galactinol	8.63 × 10^−7^	6.06	7.60 × 10^−5^	1.96 × 10^−5^	0.35
Starch and sucrose metabolism	Fructose, starch, sucrose, maltose, cellobiose, glucose, glucose‐6‐phosphate, fructose‐6‐phosphate, alpha‐trehalose	1.19 × 10^−6^	5.92	1.04 × 10^−4^	2.17 × 10^−5^	0.73

## 4 Discussion

### 4.1 Metabolic Changes in Tuber Crops Under Drought Stress

Tuber crops exhibit a wide range of metabolic responses to drought stress, with numerous studies providing valuable insights into these adaptations. In the current study, 223 metabolites were identified as being influenced by drought stress in cassava, potato and sweet potato. Some metabolites demonstrated conserved responses across the three crops, while others exhibited species‐specific changes. Notably, two metabolites (L‐proline and trehalose) were consistently reported as responsive to drought stress across the three crops reviewed in the present study.

Furthermore, the studies reviewed indicate that the metabolites accumulated in these tuber crops contribute to drought‐tolerance mechanisms, including osmotic adjustment (Fang and Xiong 2015; Zia et al. 2021). This phenomenon enables cells to maintain positive turgor pressure despite the water deficit (Zivcak et al. 2016). Osmotic adjustment involves the accumulation of osmolytes that stabilize cell structures, maintain osmotic balance, and mitigate the effects of stress (Fang and Xiong 2015; Guru et al. 2025). These osmolytes include a variety of sugars (such as glucose, fructose, sucrose and trehalose); amino acids (such as proline and gamma‐aminobutyric acid); and polyamines (such as putrescine and spermidine) (Fang and Xiong 2015). Therefore, it is important to note that metabolites also play additional roles beyond osmotic adjustment, such as antioxidant defense, signaling, energy metabolism, and membrane stabilization (Mutanda et al. 2024).

#### 4.1.1 Sugars

Sugars are among the most important osmolytes, serving dual roles as osmoprotectants and signaling molecules (Dutta et al. 2019; Ejaz et al. 2020). The comparative metabolomic analysis of cassava, potato and sweet potato revealed distinct sugars involved in plant's survival under drought‐stressed conditions (Rosa et al. 2009). The consistent detection of trehalose across all three crops suggest that it is important in their physiology. Trehalose is a well‐known stress protectant, stabilizing proteins and membranes under drought‐stressed conditions (Evers et al. 2010; Barnaby et al. 2015; Yang et al. 2015; Ren et al. 2017; Zhou et al. 2022). This suggests that all three tuber crops utilize trehalose as a core mechanism to mitigate the impact of drought stress conditions. The relatively high levels of trehalose previously observed in drought‐tolerant cassava varieties further support this hypothesis (Ren et al. 2017; Li et al. 2022). The presence of melibiose, stachyose and raffinose, which are raffinose family oligosaccharides (RFOs) involved in drought tolerance (Evers et al. 2010; Juhász et al. 2014; Yang et al. 2015; Sprenger et al. 2018; Barnaby et al. 2019), suggests that potato plants might utilize these sugars to enhance their resilience to drought stress conditions. Furthermore, the identification of maltose and starch aligns with the role of potato tubers as a major starch storage organ, with maltose being a key intermediate in starch degradation during drought stress. These findings are consistent with previous research demonstrating significant fluctuations in sugar content during potato tuber development and storage (Kumar 2011; Haider et al. 2023). Furthermore, the unique presence of lyxose, gentiobiose and erythrose in sweet potato points to distinct sugar metabolism pathways compared to cassava and potato. While the specific functions of lyxose and gentiobiose in sweet potato could be linked to cell wall metabolism. The absence of many of the potato‐specific sugars in cassava and sweet potato suggests that these crops rely on alternative mechanisms for carbohydrate storage and stress adaptation. For instance, cassava primarily stores carbohydrates as starch but may utilize different types of oligosaccharides or polyols for stress protection. Further research is needed in breeding of genotypes with manipulated genes involved in trehalose or raffinose synthesis to enhance drought tolerance in potato or cassava.

#### 4.1.2 Amino Acids

The consistent detection of proline in all three tuber crops highlights its crucial role in plant physiology, especially under drought stressed conditions (Evers et al. 2010; Yang et al. 2015; Barnaby et al. 2015; Sprenger et al. 2016; Drapal et al. 2017; Barnaby et al. 2019; Haas et al. 2020; Demirel et al. 2020; Da Ros et al. 2020; Toubiana et al. 2020; Yin, Chen, et al. 2024; Yin, Qiao, et al. 2024). This is because proline functions as an osmoprotectant which stabilizes proteins and membranes. The elevated levels of proline observed in drought‐stressed plants (Singh et al. 2015; Meena et al. 2019) further support its role in drought stress adaptation. Some studies reported a broad array of amino acids in potato, including arginine, cysteine, glutamate and lysine, highlighting their metabolic diversity under drought stress. These amino acids contribute to protein synthesis, enzyme activity and signaling processes during the period of drought stress (Ali et al. 2024). The presence of γ‐aminobutyric acid, a non‐protein amino acid with signaling functions, further enhances drought tolerance in potato cultivars (Juhász et al. 2014). The exclusive detection of L‐tyrosine, 3‐hydroxy‐L‐proline and alpha‐aminoadipic acid in sweet potato suggests distinct amino acid metabolism pathways compared to cassava and potato. The presence of these metabolites may be linked to the unique biochemical pathway enhancing the drought tolerance of sweet potato genotypes. The absence of many potato‐specific amino acids in cassava and sweet potato suggests that these crops may have lower protein content (Chandrasekara and Josheph Kumar 2016) or rely on different mechanisms for amino acid synthesis and regulation. Therefore, future research should focus on analyzing the amino acid profiles especially on different varieties of cassava to identify genetic factors that influence amino acid metabolism.

#### 4.1.3 Phenolic Acids

Phenolic acids are a class of plant secondary metabolites with diverse biological activities, including antioxidant properties. They contribute to plant defense mechanisms against oxidative stress caused by drought‐stressed conditions. The presence of 3‐phenylacetic acid and 4‐vinylphenol dimer in sweet potato points to distinct phenolic acid metabolism pathways compared to cassava and potato (Zhou et al. 2022). The presence of p‐coumaric acid, caffeic acid, ferulic acid, and sinapic acid only in potato suggests that potato may have stronger antioxidant activities than other crops (Drapal et al. 2017). Drought stress induces the production of ROS, leading to oxidative damage in plant cells. The phenolic acids can act as antioxidants that can scavenge ROS and protect cellular components from oxidative stress (Drapal et al. 2017; Sprenger et al. 2016). The presence of compounds/metabolites like 3‐phenylacetic acid in sweet potato and caffeic acid, p‐coumaric acid, ferulic acid and sinapic acid in potato suggests an active antioxidant defense mechanism in these crops. Additionally, compounds like benzoic acid, 3‐hydroxybenzoic acid and 3,4‐dihydroxybenzoic acid found in potato further enhance ROS scavenging capacity. Therefore, the identification and characterization of the phenolic acids provide valuable insights into developing crops with improved antioxidant defense mechanisms.

#### 4.1.4 Organic Acids

Organic acids are essential metabolites that play pivotal roles in plant's response to drought‐stressed conditions. In tuber crops, organic acids such as malic acid, citric acid and quinic acid accumulate under drought‐stressed conditions, contributing to osmotic adjustment and cellular homeostasis. These acids help maintain cell turgor pressure by balancing the osmotic potential within the cell, thereby preventing dehydration (Barnaby et al. 2015; Drapal et al. 2017; Haas et al. 2020). In potato, α‐ketoglutarate and quinic acid are particularly significant for their involvement in osmotic regulation, while in sweet potato, maleic acid and citraconic acid play vital roles in maintaining osmotic balance (Barnaby et al. 2015; Drapal et al. 2017; Barnaby et al. 2019; Haas et al. 2020). Beyond osmotic regulation, organic acids help buffer ion imbalances and stabilize cellular pH under drought‐stressed conditions, ensuring the optimal functioning of cellular processes (Barnaby et al. 2015; Drapal et al. 2017; Guo et al. 2018). They also serve as metabolic intermediates, acting as precursors for the biosynthesis of other essential metabolites. For instance, malic acid can be converted into sugars through gluconeogenesis, providing an alternative energy source during water scarcity (Barnaby et al. 2015; Drapal et al. 2017). In sweet potato, maleic acid and citraconic acid contribute to osmotic adjustment and modulate the activity of stress‐related enzymes, further enhancing drought tolerance (Barnaby et al. 2019; Haas et al. 2020). The presence of shared organic acids such as malic acid and citric acid in both potato and sweet potato indicates that these crops utilize common mechanisms to withstand drought stress conditions. This highlights the universal importance of organic acids in drought tolerance across different tuber crops (Barnaby et al. 2015; Drapal et al. 2017; Haas et al. 2020). Therefore, understanding the roles of organic acids and their biosynthetic pathways could provide valuable insights for breeding drought‐tolerant genotypes of cassava, potato and sweet potato.

#### 4.1.5 Polyamines

Polyamines are low‐molecular‐weight aliphatic amines that play crucial roles in various plants' physiological processes, including responses to drought‐stressed conditions (Hasan et al. 2021). The metabolites under the polyamines group (including putrescine and spermidine) stabilize the phospholipids and proteins in the cell membrane (Kapoor 2023). In addition, these metabolites are considered to have antioxidant properties (Sprenger et al. 2016) that promote the activity of antioxidant enzymes to reduce oxidative stress (Weiss and Landauer 2000). This protective role helps maintain plant growth and productivity as well as regulating stomatal conductance to reduce transpiration rate. Furthermore, polyamines play a significant role in enhancing root system architecture, which is crucial for plants to effectively absorb water and nutrients when experiencing drought stress. Research has shown that the upregulation of polyamines such as putrescine and spermidine can enhance root length, biomass, and lateral root proliferation in plants (Couée et al. 2004; Tang and Newton 2005; Tyagi et al. 2023), leading to better soil moisture utilization. Therefore, plant breeders should focus on targeting and utilizing polyamines as biomarkers for improving tuber crop root morphology, especially in cassava.

### 4.2 Metabolic Pathways Utilized to Improve Drought Tolerance of Tuber Crops

Drought stress is a major environmental constraint affecting the growth, development, and yield of tuber crops such as cassava, potato and sweet potato. These crops have developed complex metabolic strategies to cope with water stress, ensuring both survival and productivity under drought conditions (Kapoor 2023). Key adaptive mechanisms involve regulation of metabolic pathways controlling osmotic balance and stress signaling (Wasaya et al. 2021; Lal et al. 2022). Therefore, understanding how these metabolic pathways function under drought stress is essential for improving the drought resilience of tuber crops. The following sections explore the specific metabolic pathways activated in response to drought stress, highlighting the key metabolites involved and their roles in enhancing drought tolerance.

#### 4.2.1 Glyoxylate and Dicarboxylate Metabolism

The GDM pathway plays a critical role in plant survival under drought stress by facilitating energy conservation and carbon assimilation. It allows plants to use stored fatty acids as carbon sources when photosynthesis is compromised, producing essential metabolites for survival (Kunze et al. 2006; Wang, Li, and Dong 2024; Wang, Zhang, et al. 2024). This ability to generate energy from alternative sources supports plant survival and productivity, making the GDM pathway a key target for improving drought tolerance in crops. Furthermore, in plant metabolism, the glyoxylate and dicarboxylate pathway significantly upregulates the production of metabolites like malate and succinate. These metabolites act as osmolytes to maintain cell turgor and balance metabolic disruptions caused by reduced photosynthesis. The enzymes (isocitrate lyase and malate synthase) involved in this pathway are crucial for the conversion of acetyl‐CoA into succinate, a precursor for malate synthesis (Yuenyong et al. 2019) to maintain cell turgor under drought stress (Ahn et al. 2016). Therefore, these insights suggest that metabolic engineering of GDM could improve drought resilience and yield stability under water‐limited environments.

#### 4.2.2 TCA Cycle

The TCA cycle plays a vital role in enhancing drought tolerance in plants by sustaining energy production and ensuring metabolic flexibility under drought‐stressed conditions (Li, Yang, et al. 2021; Li, Shao, et al. 2021). Limited water availability reduces photosynthesis and energy supply, but tuber crops can maintain ATP production by increasing TCA cycle flux. Key intermediates including malate and succinate support mitochondrial respiration, enabling continuous energy generation. Additionally, citrate regulates carbon flow and prevents metabolic imbalances that affect growth under drought stress (Tahjib‐Ul‐Arif et al. 2021). The elevated levels of iso‐citrate and 2‐oxo‐glutarate promote the synthesis of proline and glutamate (Gai et al. 2020), which help mitigate oxidative stress induced by drought‐related ROS. Moreover, the phosphoenolpyruvate and pyruvate nodes of the TCA cycle contribute to drought resilience (Diniz et al. 2020) by facilitating carbon partitioning and metabolic adjustments. Phosphoenolpyruvate supports carbon metabolism, aiding gluconeogenesis and energy redistribution (Shi et al. 2015; Hu et al. 2025), while pyruvate connects glycolysis to the TCA cycle (Zhang and Fernie 2023), ensuring a steady supply of carbon skeletons for energy production and biosynthesis under drought. Therefore, these findings highlight the potential for targeted metabolic interventions to optimize TCA cycle flux and support drought tolerance in tuber crops.

#### 4.2.3 Alanine, Aspartate and Glutamate Metabolism

The metabolic pathway involving alanine, aspartate and glutamate is pivotal in bolstering drought tolerance in tuber crops by facilitating crucial physiological and biochemical adaptations. This pathway is central to nitrogen assimilation and redistribution processes that are indispensable for maintaining metabolic equilibrium under water‐limited conditions (Raza et al. 2025). In addition, these amino acids enhance the plant's capacity to adapt to drought stress by optimizing nitrogen utilization for protein synthesis and other vital metabolic processes. Notably, glutamate serves as a precursor for proline synthesis (Stolarz and Hanaka 2025), a key osmolyte that helps maintain cellular turgor pressure and protects proteins from denaturation under drought stress. Aspartate and alanine mitigate oxidative damage by supporting pathways linked to cellular homeostasis under drought conditions (Wang, Li, and Dong 2024; Wang, Zhang, et al. 2024). Furthermore, these amino acids act as signaling molecules, modulating stress responses and adaptive mechanisms in plants (Raza et al. 2024). Therefore, these amino acids reinforce plant drought tolerance through both biochemical and signaling roles.

#### 4.2.4 Galactose Metabolism

Drought stress significantly activates the galactose metabolism in plants to enhance survival and productivity (Zhang, Yan, et al. 2021; Zhang, Luan, et al. 2021). Under drought stressed conditions, tuber crops accumulate metabolites including stachyose, raffinose and galactinol, which act as osmoprotectants to prevent oxidative damage (Nishizawa et al. 2008; Sanyal et al. 2023). The myo‐inositol is an important metabolite in this pathway, which enhances drought tolerance by regulating stomatal closure and helps maintain water balance during drought stress (Hu et al. 2022). These metabolic adjustments collectively aid in preserving cell turgor and mitigating the physiological stress associated with water deficit. The galactose metabolism integrates with carbohydrate metabolism to help plants adapt to drought stress (Gundaraniya et al. 2020). Furthermore, when water availability declines, sucrose (from the galactose metabolism) is hydrolysed into glucose and fructose (Bagherikia et al. 2019), providing immediate energy to sustain cellular activities ensuring tuber crop survival during drought stress. The specific metabolites within galactose metabolism, such as melibiose and glycerol, enhance osmoprotection, which allows tuber crops to efficiently adapt to water stress. These adaptations highlight the critical role of galactose metabolism in improving drought resilience and offering potential for metabolic engineering aimed at enhancing drought tolerance in tuber crops.

#### 4.2.5 Starch and Sucrose Metabolism

Drought stress significantly impacts the starch and sucrose metabolism of tuber crops, leading to the alteration of sugars in tuber crops (Geigenberger et al. 1997). During drought stress, plants tend upregulate sugars such as sucrose in tuber crops (Kaur et al. 2021) due to reduced activities of sucrose synthase, which converts sucrose into glucose and fructose. This results in decreased starch synthesis as enzymes crucial for starch biosynthesis, like ADP‐glucose pyrophosphorylase and starch synthase. Consequently, starch storage in tubers is impaired lead to poor tuber development and tuber yield (Ahmad et al. 2024). The intermediate metabolites such as glucose‐6‐phosphate and fructose‐6‐phosphate essential for starch biosynthesis decline under drought stressed conditions due to reduced photosynthetic rate (Wang, Liu, et al. 2022; Wang, Yang, et al. 2022; Thomas and Beena 2024). Furthermore, the accumulation of sucrose and its derivatives, such as alpha‐trehalose, plays a crucial role in protecting cell membranes and proteins from dehydration‐induced damage (Benaroudj and Goldberg 2001). These integrated metabolic responses optimize carbohydrate utilization, reinforcing tuber crop resilience under drought conditions. Therefore, a deeper understanding of this metabolic pathway will assist in enhancing drought tolerance tuber crop cultivars.

## 5 Summary

The study identified a wide range of metabolites including sugars (trehalose and sucrose), amino acids (proline and glutamate) and organic acids (malic acid and citric acid) which were altered under drought stressed conditions. These metabolites play crucial roles in osmotic regulation, oxidative stress management and energy conservation, enhancing drought tolerance mechanisms in tuber crops. The review highlighted the importance of specific metabolic pathways, including the GDM, citrate cycle and galactose metabolism, which contribute to the adaptive responses of tuber crops to water stress conditions. While the current understanding of the metabolomic responses to drought stress in these crops is significant, it remains fragmented. Further studies are necessary to address the inconsistencies in metabolite profiles across different studies and crops. In particular, the regulatory mechanisms of cassava need to be thoroughly dissected to understand its ability to withstand harsh climatic conditions including drought stress, as well as the adeptness to thrive in marginal soils. The new understanding of the crop's resilience can be adopted and help breeders in developing a wide range of climate‐smart crops for agricultural sustainability and achieving food security.

## 6 Way Forward

1. Breeding programs should focus on enhancing the production of key metabolites such as proline, trehalose and polyamines, which play critical roles in drought tolerance.
2. The key metabolic pathways involved in drought tolerance, such as the GDM, citrate cycle and galactose metabolism should be explored further through metabolic engineering to improve drought resilience in tuber crops.
3. Future research should focus on standardized methodologies and experimental conditions to ensure the reproducibility of results across different studies.
4. Given their protective roles in osmotic regulation, antioxidant activity and root architecture, polyamines should be explored as potential biomarkers for improving root system efficiency and water utilization under drought stress conditions.
5. Integrating metabolomics with transcriptomics and proteomics could provide a more comprehensive overview of tuber crop responses to drought stress.

## Funding

We would like to thank the Water Research Commission for funding this research. This paper is based on research under the project titled “An integrated multi‐omics approach to uncover drought tolerance biomarkers in two underutilised crops: sweet potato ( *Ipomoea batatas* L.) and cassava ( *Manihot esculenta* Crantz)” funded by the Water Research Commission (Project No. C2023/2024–01262), with grant funds awarded and managed by the University of South Africa (UNISA). The contents of the article are the authors' sole responsibility and should not be regarded as reflecting the views and position of the WRC and UNISA.

## Conflicts of Interest

The authors declare no conflicts of interest.

## Supporting information


**Table S1:** List of significantly changed metabolites in selected three major tuber crops under drought stress conditions.
**Figure S1:** Pathway analysis using all identified metabolites in major tuber crops showing metabolic pathways represented as nodes. The graph presents a view of all the matched pathways arranged by *p* values on the *y*‐axis and the pathway impact values on the *x*‐axis. The node color (beige to red) is based on the node's *p* value, and the node radius is defined by the pathway impact values. A pathway impact value > 0.1 and *p* < 0.05 was considered a target.

## Data Availability

Data underlying the findings of this study are available in the Supporting Information file.

## References

[pei370126-bib-0001] Ahmad, D. , Y. Ying , and J. Bao . 2024. “Understanding Starch Biosynthesis in Potatoes for Metabolic Engineering to Improve Starch Quality: A Detailed Review.” Carbohydrate Polymers 346: 122592. 39245484 10.1016/j.carbpol.2024.122592

[pei370126-bib-0002] Ahn, S. , J. Jung , I. A. Jang , E. L. Madsen , and W. Park . 2016. “Role of Glyoxylate Shunt in Oxidative Stress Response.” Journal of Biological Chemistry 291, no. 22: 11928–11938. 27036942 10.1074/jbc.M115.708149 PMC4882458

[pei370126-bib-0003] Ali, H. , I. Mahmood , M. F. Ali , et al. 2024. “Individual and Interactive Effects of Amino Acid and Paracetamol on Growth, Physiological and Biochemical Aspects of *Brassica napus* L. Under Drought Conditions.” Heliyon 10, no. 11: e31544. 38882271 10.1016/j.heliyon.2024.e31544 PMC11176763

[pei370126-bib-0004] Almeida, T. , G. Pinto , B. Correia , S. Gonçalves , M. Meijon , and M. Escandón . 2020. “In‐Depth Analysis of the *Quercus suber* Metabolome Under Drought Stress and Recovery Reveals Potential Key Metabolic Players.” Plant Science 299: 110606. 32900444 10.1016/j.plantsci.2020.110606

[pei370126-bib-0005] Arbona, V. , M. Manzi , C. de Ollas , and A. Gómez‐Cadenas . 2013. “Metabolomics as a Tool to Investigate Abiotic Stress Tolerance in Plants.” International Journal of Molecular Sciences 14, no. 3: 4885–4911. 23455464 10.3390/ijms14034885 PMC3634444

[pei370126-bib-0007] Bagherikia, S. , M. Pahlevani , A. Yamchi , K. Zaynalinezhad , and A. Mostafaie . 2019. “Transcript Profiling of Genes Encoding Fructan and Sucrose Metabolism in Wheat Under Terminal Drought Stress.” Journal of Plant Growth Regulation 38: 148–163.

[pei370126-bib-0008] Barnaby, J. Y. , D. Fleisher , V. Reddy , and R. Sicher . 2015. “Combined Effects of CO_2_ Enrichment, Diurnal Light Levels and Water Stress on Foliar Metabolites of Potato Plants Grown in Naturally Sunlit Controlled Environment Chambers.” Physiologia Plantarum 153, no. 2: 243–252. 24888746 10.1111/ppl.12238

[pei370126-bib-0009] Barnaby, J. Y. , D. H. Fleisher , S. K. Singh , R. C. Sicher , and V. R. Reddy . 2019. “Combined Effects of Drought and CO2 Enrichment on Foliar Metabolites of Potato (*Solanum tuberosum* L.) Cultivars.” Journal of Plant Interactions 14, no. 1: 110–118.

[pei370126-bib-0010] Begna, T. 2021. “Impact of Drought Stress on Crop Production and Its Management Options.” International Journal of Research in Agronomy 8, no. 2: 1–13.

[pei370126-bib-0011] Benaroudj, N. , and A. L. Goldberg . 2001. “Trehalose Accumulation During Cellular Stress Protects Cells and Cellular Proteins From Damage by Oxygen Radicals.” Journal of Biological Chemistry 276, no. 26: 24261–24267. 11301331 10.1074/jbc.M101487200

[pei370126-bib-0012] Burnett, A. C. , S. P. Serbin , K. J. Davidson , K. S. Ely , and A. Rogers . 2021. “Detection of the Metabolic Response to Drought Stress Using Hyperspectral Reflectance.” Journal of Experimental Botany 72, no. 18: 6474–6489. 34235536 10.1093/jxb/erab255

[pei370126-bib-0013] Chandrasekara, A. , and T. Josheph Kumar . 2016. “Roots and Tuber Crops as Functional Foods: A Review on Phytochemical Constituents and Their Potential Health Benefits.” International Journal of Food Science 2016: 3631647. 10.1155/2016/3631647. 27127779 PMC4834168

[pei370126-bib-0151] Chauhan, V. B. S. , S. N. Mallick , K. Pati , R. Arutselvan , and M. Nedunchezhiyan . 2022. “Status and Importance of Underexploited Tuber Crops in Relation to Nutritional Security and Economic Prosperity.” In Compendium for Winter School on “Unexpected Vegetables: Unexplored Treasure Trove for Food”, 246–264. ICAR‐Indian Institute of Vegetable Research: Varanasi, India.

[pei370126-bib-0016] Couée, I. , I. Hummel , C. Sulmon , G. Gouesbet , and A. El Amrani . 2004. “Involvement of Polyamines in Root Development.” Plant Cell, Tissue and Organ Culture 76: 1–10.

[pei370126-bib-0017] Cruz de Carvalho, M. H. 2008. “Drought Stress and Reactive Oxygen Species: Production, Scavenging and Signaling.” Plant Signaling & Behavior 3, no. 3: 156–165. 19513210 10.4161/psb.3.3.5536 PMC2634109

[pei370126-bib-0018] Da Ros, L. , R. Elferjani , R. Soolanayakanahally , et al. 2020. “Drought‐Induced Regulatory Cascades and Their Effects on the Nutritional Quality of Developing Potato Tubers.” Genes 11, no. 8: 864. 10.3390/genes11080864. 32751417 PMC7465940

[pei370126-bib-0019] Demirel, U. , W. L. Morris , L. J. M. Ducreux , et al. 2020. “Physiological, Biochemical, and Transcriptional Responses to Single and Combined Abiotic Stress in Stress‐Tolerant and Stress‐Sensitive Potato Genotypes.” Frontiers in Plant Science 11: 169. 10.3389/fpls.2020.00169. 32184796 PMC7058966

[pei370126-bib-0020] Diniz, A. L. , D. I. R. da Silva , C. G. Lembke , et al. 2020. “Amino Acid and Carbohydrate Metabolism Are Coordinated to Maintain Energetic Balance During Drought in Sugarcane.” International Journal of Molecular Sciences 21, no. 23: 9124. 10.3390/ijms21239124. 33266228 PMC7729667

[pei370126-bib-0024] Drapal, M. , E. R. Farfan‐Vignolo , O. R. Gutierrez , M. Bonierbale , E. Mihovilovich , and P. D. Fraser . 2017. “Identification of Metabolites Associated With Water Stress Responses in *Solanum tuberosum* L. Clones.” Phytochemistry 135: 24–33. 10.1016/j.phytochem.2016.12.003. 27964835

[pei370126-bib-0025] Dutta, T. , N. R. R. Neelapu , S. H. Wani , and C. Surekha . 2019. “Role and Regulation of Osmolytes as Signaling Molecules to Abiotic Stress Tolerance.” In Plant Signaling Molecules, 459–477. Elsevier.

[pei370126-bib-0026] Ejaz, S. , S. Fahad , M. A. Anjum , et al. 2020. “Role of Osmolytes in the Mechanisms of Antioxidant Defense of Plants.” Sustainable Agriculture Reviews 39: 95–117.

[pei370126-bib-0028] Evers, D. , I. Lefèvre , S. Legay , et al. 2010. “Identification of Drought‐Responsive Compounds in Potato Through a Combined Transcriptomic and Targeted Metabolite Approach.” Journal of Experimental Botany 61, no. 9: 2327–2343. 10.1093/jxb/erq060. 20406784

[pei370126-bib-0030] Fang, Y. , and L. Xiong . 2015. “General Mechanisms of Drought Response and Their Application in Drought Resistance Improvement in Plants.” Cellular and Molecular Life Sciences 72: 673–689. 25336153 10.1007/s00018-014-1767-0 PMC11113132

[pei370126-bib-0032] Gai, Z. , L. Liu , J. Zhang , J. Liu , and L. Cai . 2020. “Effects of Exogenous α‐Oxoglutarate on Proline Accumulation, Ammonium Assimilation and Photosynthesis of Soybean Seedling (*Glycine max* (L.) Merr.) Exposed to Cold Stress.” Scientific Reports 10: 17017. 33046814 10.1038/s41598-020-74094-w PMC7550343

[pei370126-bib-0034] Geigenberger, P. , R. Reimholz , M. Geiger , L. Merlo , V. Canale , and M. Stitt . 1997. “Regulation of Sucrose and Starch Metabolism in Potato Tubers in Response to Short‐Term Water Deficit.” Planta 201: 502–518.

[pei370126-bib-0035] Ghosh, U. K. , M. N. Islam , M. N. Siddiqui , X. Cao , and M. A. R. Khan . 2022. “Proline, a Multifaceted Signalling Molecule in Plant Responses to Abiotic Stress: Understanding the Physiological Mechanisms.” Plant Biology 24: 227–239. 34796604 10.1111/plb.13363

[pei370126-bib-0036] Gundaraniya, S. A. , P. S. Ambalam , and R. S. Tomar . 2020. “Metabolomic Profiling of Drought‐Tolerant and Susceptible Peanut ( *Arachis hypogaea* L.) Genotypes in Response to Drought Stress.” ACS Omega 5: 31209–31219. 33324830 10.1021/acsomega.0c04601 PMC7726923

[pei370126-bib-0037] Guo, R. , L. Shi , Y. Jiao , et al. 2018. “Metabolic Responses to Drought Stress in the Tissues of Drought‐Tolerant and Drought‐Sensitive Wheat Genotype Seedlings.” AoB Plants 10: ply016. 29623182 10.1093/aobpla/ply016 PMC5881611

[pei370126-bib-0038] Guru, A. , D. Ranjan , D. Jaiswal , A. Hidangmayum , and P. Dwivedi . 2025. “Role of Osmolytes in ROS‐Scavenging and Maintaining Homeostasis Under Abiotic Stress.” In Plant Signaling Molecules in Regulation of ROS‐Scavenging System: Adapting to Climate Change, 61–86. Springer Nature Singapore.

[pei370126-bib-0039] Haas, M. , H. Sprenger , E. Zuther , et al. 2020. “Can Metabolite‐ and Transcript‐Based Selection for Drought Tolerance in *Solanum tuberosum* Replace Selection on Yield in Arid Environments?” Frontiers in Plant Science 11: 1071. 10.3389/fpls.2020.01071. 32793257 PMC7385397

[pei370126-bib-0040] Haider, M. W. , M. Nafees , R. Iqbal , et al. 2023. “Postharvest Starch and Sugars Adjustment in Potato Tubers of Wide‐Ranging Dormancy Genotypes Subjected to Various Sprout Forcing Techniques.” Scientific Reports 13: 14845. 37684294 10.1038/s41598-023-37711-y PMC10491617

[pei370126-bib-0041] Hasan, M. M. , M. Skalicky , M. S. Jahan , et al. 2021. “Spermine: Its Emerging Role in Regulating Drought Stress Responses in Plants.” Cells 10: 261. 33525668 10.3390/cells10020261 PMC7912026

[pei370126-bib-0045] Hu, L. Y. , Y. U. E. Hong , J. Y. Zhang , L. I. Yang‐tian‐su , X. Q. Gong , and Z. H. O. U. Kun . 2022. “Overexpression of MdMIPS1 Enhances Drought Tolerance and Water‐Use Efficiency in Apple.” Journal of Integrative Agriculture 21: 1968–1981.

[pei370126-bib-0046] Hu, R. , H. Yu , J. Deng , et al. 2025. “Phosphoenolpyruvate and Related Metabolic Pathways Contribute to the Regulation of Plant Growth and Development.” International Journal of Molecular Sciences 26: 391. 39796250 10.3390/ijms26010391 PMC11720000

[pei370126-bib-0049] Juhász, Z. , D. Balmer , A. Sós‐Hegedûs , A. Vallat , B. Mauch‐Mani , and Z. Bánfalvi . 2014. “Effects of Drought Stress and Storage on the Metabolite and Hormone Contents of Potato Tubers Expressing the Yeast Trehalose‐6‐Phosphate Synthase 1 Gene.” Journal of Agricultural Science 6: 142.

[pei370126-bib-0050] Kapoor, R. T. 2023. “Role of Polyamines in Plants Under Abiotic Stresses: Regulation of Biochemical Interactions.” Plant Stress Mitigators 2023: 209–220.

[pei370126-bib-0051] Kaur, H. , M. Manna , T. Thakur , V. Gautam , and P. Salvi . 2021. “Imperative Role of Sugar Signaling and Transport During Drought Stress Responses in Plants.” Physiologia Plantarum 171: 833–848. 33583052 10.1111/ppl.13364

[pei370126-bib-0052] Khamis, G. , E. A. Alsherif , S. M. Korany , et al. 2025. “Drought Stress Differentially Influences Growth, Physiology, and Metabolite Accumulation in *Triticum aestivum* (C3) and *Amaranthus caudatus* (C4) Plants.” BMC Plant Biology 25, no. 1: 1199. 40926199 10.1186/s12870-025-07022-7 PMC12418684

[pei370126-bib-0055] Kumar, D. 2011. “Cold‐Induced Sweetening Development in Indian Potato ( *Solanum tuberosum* L.) Varieties.” Indian Journal of Biochemistry & Biophysics 48: 123–127. 21682144

[pei370126-bib-0057] Kunze, M. , I. Pracharoenwattana , S. M. Smith , and A. Hartig . 2006. “A Central Role for the Peroxisomal Membrane in Glyoxylate Cycle Function.” Biochimica et Biophysica Acta 1763, no. 12: 1441–1452. 17055076 10.1016/j.bbamcr.2006.09.009

[pei370126-bib-0058] Lal, M. K. , R. K. Tiwari , A. Kumar , et al. 2022. “Mechanistic Concept of Physiological, Biochemical, and Molecular Responses of the Potato Crop to Heat and Drought Stress.” Plants 11, no. 21: 2857. 36365310 10.3390/plants11212857 PMC9654185

[pei370126-bib-0060] Li, P. C. , X. Y. Yang , H. M. Wang , et al. 2021. “Metabolic Responses to Combined Water Deficit and Salt Stress in Maize Primary Roots.” Journal of Integrative Agriculture 20, no. 1: 109–119.

[pei370126-bib-0061] Li, S. , Z. Shao , C. Lu , J. Yao , Y. Zhou , and D. Duan . 2021. “Glutamate Dehydrogenase Functions in Glutamic Acid Metabolism and Stress Resistance in *Pyropia haitanensis* .” Molecules 26, no. 22: 6793. 34833887 10.3390/molecules26226793 PMC8623670

[pei370126-bib-0063] Li, Z. B. , S. M. Dong , C. Y. Zeng , P. J. Zhao , S. X. Li , and M. Peng . 2022. “Correlation Between Soluble Sugar and Tolerance to Drought Stress of Cassava Stem Under Low Temperature Storage.” Journal of South China Agricultural University 43: 58–66. 10.7671/j.issn.1001-411X.202109030.

[pei370126-bib-0065] Makhubu, F. N. , M. Mutanda , N. E. Madala , and S. Figlan . 2024. “Metabolite Profiling in Ten Bread Wheat (*Triticum aestivum* L.) Genotypes in Response to Drought Stress.” Plant Stress 14: 100680.

[pei370126-bib-0066] Makhubu, F. N. , L. E. Siviya , M. E. Rauwane , S. M. Laurie , N. E. Madala , and S. Figlan . 2025. “Biochemical Responses of Atacama and Blesbok Sweet Potato (*Ipomoea batatas* L.) Cultivars to Early Drought Stress.” Plants 14: 3532. 41304683 10.3390/plants14223532 PMC12655927

[pei370126-bib-0070] Meena, M. , K. Divyanshu , S. Kumar , et al. 2019. “Regulation of L‐Proline Biosynthesis, Signal Transduction, Transport, Accumulation and Its Vital Role in Plants During Variable Environmental Conditions.” Heliyon 5, no. 12: e02952. 31872123 10.1016/j.heliyon.2019.e02952 PMC6909094

[pei370126-bib-0074] Mutanda, M. , A. B. Amelework , N. Ndou , and S. Figlan . 2025. “Drought Stress in Cassava (*Manihot esculenta*): Management Strategies and Breeding Technologies.” International Journal of Plant Biology 16: 112.

[pei370126-bib-0075] Mutanda, M. , S. Figlan , V. Chaplot , N. E. Madala , and H. Shimelis . 2024. “Selection of Wheat (*Triticum aestivum* L.) Genotypes Using Yield Components, Water Use Efficiency and Major Metabolites Under Drought Stress.” Journal of Agronomy and Crop Science 210: e12766.

[pei370126-bib-0076] Mutanda, M. , H. Shimelis , V. Chaplot , and S. Figlan . 2025. “Managing Drought Stress in Wheat (*Triticum aestivum* L.) Production: Strategies and Impacts.” South African Journal of Plant and Soil 42: 1–12.

[pei370126-bib-0152] Nanbol, K. K. , and O. Namo . 2019. “The Contribution of Root and Tuber Crops to Food Security: A Review.” Journal of Agricultural Science and Technology B 9, no. 4. 10.17265/2161-6264/2019.04.001.

[pei370126-bib-0079] Nishizawa, A. , Y. Yabuta , and S. Shigeoka . 2008. “Galactinol and Raffinose Constitute a Novel Function to Protect Plants From Oxidative Damage.” Plant Physiology 147, no. 3: 1251–1263. 18502973 10.1104/pp.108.122465 PMC2442551

[pei370126-bib-0080] Obata, T. , P. A. Klemens , L. Rosado‐Souza , et al. 2020. “Metabolic Profiles of Six African Cultivars of Cassava (*Manihot esculenta* Crantz) Highlight Bottlenecks of Root Yield.” Plant Journal 102, no. 6: 1202–1219. 10.1111/tpj.14693 31950549

[pei370126-bib-0082] Page, M. J. , J. E. McKenzie , P. M. Bossuyt , et al. 2021. “The PRISMA 2020 Statement: An Updated Guideline for Reporting Systematic Reviews.” BMJ 372: n71. 33782057 10.1136/bmj.n71 PMC8005924

[pei370126-bib-0087] Qiu, S. , Y. Cai , H. Yao , et al. 2023. “Small Molecule Metabolites: Discovery of Biomarkers and Therapeutic Targets.” Signal Transduction and Targeted Therapy 8, no. 1: 132. 36941259 10.1038/s41392-023-01399-3 PMC10026263

[pei370126-bib-0090] Raza, A. , M. Anas , S. Bhardwaj , et al. 2025. “Harnessing Metabolomics for Enhanced Crop Drought Tolerance.” Crop Journal 13: 311–327.

[pei370126-bib-0091] Raza, A. , S. Bhardwaj , M. A. Rahman , et al. 2024. “Fighting to Thrive via Plant Growth Regulators: Green Chemical Strategies for Drought Stress Tolerance.” Physiologia Plantarum 176, no. 6: e14605. 39513406 10.1111/ppl.14605

[pei370126-bib-0092] Ren, M. Y. , R. J. Feng , H. R. Shi , et al. 2017. “Expression Patterns of Members of the Ethylene Signaling–Related Gene Families in Response to Dehydration Stresses in Cassava.” PLoS One 12, no. 5: e0177621. 28542282 10.1371/journal.pone.0177621 PMC5441607

[pei370126-bib-0095] Rosa, M. , C. Prado , G. Podazza , et al. 2009. “Soluble Sugars: Metabolism, Sensing and Abiotic Stress: A Complex Network in the Life of Plants.” Plant Signaling & Behavior 4, no. 5: 388–393. 19816104 10.4161/psb.4.5.8294 PMC2676748

[pei370126-bib-0096] Rosado‐Souza, L. , L. C. David , M. Drapal , et al. 2019. “Cassava Metabolomics and Starch Quality.” Current Protocols in Plant Biology 4, no. 4: e20102. 31834991 10.1002/cppb.20102

[pei370126-bib-0098] Sachdev, S. , S. A. Ansari , M. I. Ansari , M. Fujita , and M. Hasanuzzaman . 2021. “Abiotic Stress and Reactive Oxygen Species: Generation, Signaling, and Defense Mechanisms.” Antioxidants 10, no. 2: 277. 33670123 10.3390/antiox10020277 PMC7916865

[pei370126-bib-0102] Sanyal, R. , S. Kumar , A. Pattanayak , A. Kar , and S. K. Bishi . 2023. “Optimizing Raffinose Family Oligosaccharides Content in Plants: A Tightrope Walk.” Frontiers in Plant Science 14: 1134754. 37056499 10.3389/fpls.2023.1134754 PMC10088399

[pei370126-bib-0105] Shah, W. , and S. Z. Satti . 2023. “Physiological, Biochemical and Morphological Responses of Plants to Water Deficit Conditions: A Review.” Pakistan Journal of Forestry 73, no. 2: 62–69.

[pei370126-bib-0108] Shi, J. , K. Yi , Y. Liu , et al. 2015. “Phospho Enol Pyruvate Carboxylase in Arabidopsis Leaves Plays a Crucial Role in Carbon and Nitrogen Metabolism.” Plant Physiology 167, no. 3: 671–681. 25588735 10.1104/pp.114.254474 PMC4348777

[pei370126-bib-0111] Singh, M. , J. Kumar , S. Singh , V. P. Singh , and S. M. Prasad . 2015. “Roles of Osmoprotectants in Improving Salinity and Drought Tolerance in Plants: A Review.” Reviews in Environmental Science and Bio/Technology 14: 407–426.

[pei370126-bib-0112] Sprenger, H. , A. Erban , S. Seddig , et al. 2018. “Metabolite and Transcript Markers for the Prediction of Potato Drought Tolerance.” Plant Biotechnology Journal 16, no. 4: 939–950. 10.1111/pbi.12840. 28929574 PMC5866952

[pei370126-bib-0113] Sprenger, H. , C. Kurowsky , R. Horn , et al. 2016. “The Drought Response of Potato Reference Cultivars With Contrasting Tolerance.” Plant, Cell & Environment 39, no. 11: 2370–2389. 10.1111/pce.12780 27341794

[pei370126-bib-0114] Stolarz, M. , and A. Hanaka . 2025. “Glutamate and Its Role in the Metabolism of Plants and Animals.” Processes 13: 2084.

[pei370126-bib-0115] Tahjib‐Ul‐Arif, M. , M. I. Zahan , M. M. Karim , et al. 2021. “Citric Acid‐Mediated Abiotic Stress Tolerance in Plants.” International Journal of Molecular Sciences 22, no. 13: 7235. 34281289 10.3390/ijms22137235 PMC8268203

[pei370126-bib-0116] Tang, W. , and R. J. Newton . 2005. “Polyamines Promote Root Elongation and Growth by Increasing Root Cell Division in Regenerated Virginia Pine ( *Pinus virginiana* Mill.) Plantlets.” Plant Cell Reports 24: 581–589. 16160835 10.1007/s00299-005-0021-5

[pei370126-bib-0117] Thomas, A. , and R. Beena . 2024. “Sucrose Metabolism in Plants Under Drought Stress Condition: A Review.” Indian Journal of Agricultural Research 58: 943–952.

[pei370126-bib-0118] Toubiana, D. , R. Cabrera , E. Salas , et al. 2020. “Morphological and Metabolic Profiling of a Tropical‐Adapted Potato Association Panel Subjected to Water Recovery Treatment Reveals New Insights Into Plant Vigor.” Plant Journal 103, no. 6: 2193–2210. 10.1111/tpj.14892. PMC7540292 32579242

[pei370126-bib-0120] Tyagi, A. , S. Ali , G. Ramakrishna , et al. 2023. “Revisiting the Role of Polyamines in Plant Growth and Abiotic Stress Resilience: Mechanisms, Crosstalk, and Future Perspectives.” Journal of Plant Growth Regulation 42, no. 8: 5074–5098.

[pei370126-bib-0123] Wang, X. , X. Li , and S. Dong . 2024. “Biochemical Characterization and Metabolic Reprogramming of Amino Acids in Soybean Roots Under Drought Stress.” Physiologia Plantarum 176, no. 3: e14319. 38693848 10.1111/ppl.14319

[pei370126-bib-0124] Wang, X. , H. Liu , D. Zhang , et al. 2022. “Photosynthetic Carbon Fixation and Sucrose Metabolism Supplemented by Weighted Gene Co‐Expression Network Analysis in Response to Water Stress in Rice With Overlapping Growth Stages.” Frontiers in Plant Science 13: 864605. 35528941 10.3389/fpls.2022.864605 PMC9069116

[pei370126-bib-0126] Wang, Y. , Y. Zhang , H. Qiao , Y. Zheng , X. Hou , and L. Shi . 2024. “An Integrated Transcriptome and Physiological Analysis of Nitrogen Use Efficiency in Rice (*Oryza sativa* L. ssp. *Indica*) Under Drought Stress.” Frontiers in Genetics 15: 1483113. 39553474 10.3389/fgene.2024.1483113 PMC11564168

[pei370126-bib-0127] Wang, Z. , Y. Yang , V. Yadav , et al. 2022. “Drought‐Induced Proline Is Mainly Synthesized in Leaves and Transported to Roots in Watermelon Under Water Deficit.” Horticultural Plant Journal 8, no. 5: 615–626.

[pei370126-bib-0128] Wasaya, A. , S. Manzoor , T. A. Yasir , et al. 2021. “Evaluation of Fourteen Bread Wheat (*Triticum aestivum* L.) Genotypes by Observing Gas Exchange Parameters, Relative Water and Chlorophyll Content, and Yield Attributes Under Drought Stress.” Sustainability 13, no. 9: 4799.

[pei370126-bib-0132] Weiss, J. F. , and M. R. Landauer . 2000. “Radioprotection by Antioxidants a.” Annals of the New York Academy of Sciences 899, no. 1: 44–60. 10863528

[pei370126-bib-0135] Yang, J. Y. , D. H. Fleisher , R. C. Sicher , J. Kim , V. C. Baligar , and V. R. Reddy . 2015. “Effects of CO2 Enrichment and Drought Pretreatment on Metabolite Responses to Water Stress and Subsequent Rehydration Using Potato Tubers From Plants Grown in Sunlit Chambers.” Journal of Plant Physiology 189: 126–136. 10.1016/j.jplph.2015.10.004. 26600557

[pei370126-bib-0136] Yin, R. , L. Chen , P. Deng , X. Cao , and X. Xu . 2024. “Characterization of Changes in Active Ingredients and Mining of Key Metabolites in *Bletilla striata* Under Shading and Drought Stresses.” Horticulturae 10, no. 2: 163.

[pei370126-bib-0137] Yin, Y. , S. Qiao , Z. Kang , et al. 2024. “Transcriptome and Metabolome Analyses Reflect the Molecular Mechanism of Drought Tolerance in Sweet Potato.” Plants 13, no. 3: 351. 38337884 10.3390/plants13030351 PMC10857618

[pei370126-bib-0141] Yuenyong, W. , S. Sirikantaramas , L. J. Qu , and T. Buaboocha . 2019. “Isocitrate Lyase Plays Important Roles in Plant Salt Tolerance.” BMC Plant Biology 19: 1–14. 31694539 10.1186/s12870-019-2086-2 PMC6833277

[pei370126-bib-0142] Zhang, L. , S. Yan , S. Zhang , P. Yan , J. Wang , and H. Zhang . 2021. “Glutathione, Carbohydrate and Other Metabolites of *Larix olgensis* A. Henry Response to Polyethylene Glycol‐Simulated Drought Stress.” PLoS One 16, no. 11: e0253780. 34788320 10.1371/journal.pone.0253780 PMC8598043

[pei370126-bib-0143] Zhang, Y. , and A. R. Fernie . 2023. “The Role of TCA Cycle Enzymes in Plants.” Advanced Biology 7, no. 8: 2200238. 10.1002/adbi.202200238 37341441

[pei370126-bib-0144] Zhang, Y. , Q. Luan , J. Jiang , and Y. Li . 2021. “Prediction and Utilization of Malondialdehyde in Exotic Pine Under Drought Stress Using Near‐Infrared Spectroscopy.” Frontiers in Plant Science 12: 735275. 34733301 10.3389/fpls.2021.735275 PMC8558207

[pei370126-bib-0146] Zhou, Z. , J. Tang , Q. Cao , Z. Li , and D. Ma . 2022. “Differential Response of Physiology and Metabolic Response to Drought Stress in Different Sweetpotato Cultivars.” PLoS One 17, no. 3: e0264847. 35271628 10.1371/journal.pone.0264847 PMC8912141

[pei370126-bib-0148] Zia, R. , M. S. Nawaz , M. J. Siddique , S. Hakim , and A. Imran . 2021. “Plant Survival Under Drought Stress: Implications, Adaptive Responses, and Integrated Rhizosphere Management Strategy for Stress Mitigation.” Microbiological Research 242: 126626. 33189069 10.1016/j.micres.2020.126626

[pei370126-bib-0149] Zivcak, M. , M. Brestic , and O. Sytar . 2016. “Osmotic Adjustment and Plant Adaptation to Drought Stress. Drought Stress Tolerance in Plants.” Physiology and Biochemistry 1: 105–143.

