# Supplementary material for: Role of Altered Metabolites and Metabolic Pathways in Major Tuber Crops Under Drought Stress
Source: Plant Environ Interact. 2026 Feb 13;7(1):e70126. doi: 10.1002/pei3.70126 (PMC12905506; doi:10.1002/pei3.70126)
Supplement: Supplementary file 1 — Table S1: List of significantly changed metabolites in selected three major tuber crops under drought stress conditions. Figure S1: Pathway analysis using all identified metabolites in major tuber crops showing metabolic pathways represented as nodes. The graph presents a view of all the matched pathways arranged by p values on the y‐axis and the pathway impact values on the x‐axis. The node color (beige to red) is based on the node's p value, and the node radius is defined by the pathway impact values. A pathway impact value > 0.1 and p < 0.05 was considered a target. [file PEI3-7-e70126-s001.docx]

**Role of Altered Metabolites and Metabolic Pathways in Major Tuber Crops under Drought Stress**

Maltase Mutanda^1^, Fikile N Makhubu, Sandiswa Figlan^1*^,

^1^Department of Agriculture and Animal Health, University of South Africa, Florida 1709, South Africa.

*Corresponding author, email: [figlas@unisa.ac.za](mailto:figlas@unisa.ac.za)

Table S1: List of significantly changed metabolites in selected three major tuber crops under drought stress conditions.

| Metabolites | Class | Cassava | Potato | Sweet Potato | References |
| --- | --- | --- | --- | --- | --- |
| Trehalose | Sugar | ✔ | ✔ | ✔ | Evers et al., 2010; Yang et al., 2015; Barnaby et al., 2015; Ren et al., 2017; Zhou et al., 2022; Li et al., 2022 |
| 4-Aminobutyric acid | Amino acid |  |  | ✔ | Juhász et al., 2014 |
| γ-aminobutyric acid (GABA) | Amino acid |  | ✔ |  | Juhász et al., 2014; Barnaby et al., 2015, 2019; Yang et al., 2015 |
| L-Tyrosine | Amino acid |  |  | ✔ | Yin et al., 2024 |
| Arginine | Amino acid |  | ✔ |  | Da Ros et al., 2020; Orsák et al., 2021 |
| Cysteine | Amino acid |  | ✔ |  | Da Ros et al., 2020 |
| Choline | Vitamin |  | ✔ |  | Toubiana et al., 2020 |
| Allantoate | Organic acid |  | ✔ |  | Drapal et al., 2017; Evers et al., 2010 |
| Arabinose | Sugar |  | ✔ |  | Drapal et al., 2017; Evers et al., 2010; Haas et al., 2020; Sprenger et al., 2016 |
| Xylose | Sugar |  | ✔ |  | Zhou et al., 2022 |
| Lxyose | Sugar |  |  | ✔ | Zhou et al., 2022 |
| DL-Anabasine | Alkaloid |  |  | ✔ | Zhou et al., 2022 |
| 3-hydroxy-L-proline | Amino acid |  |  | ✔ | Zhou et al., 2022 |
| 3-Phenylacetic acid | Phenolic acid |  |  | ✔ | Zhou et al., 2022 |
| maleic acid | Organic acid |  |  | ✔ | Zhou et al., 2022 |
| Gentiobiose | Sugar |  |  | ✔ | Zhou et al., 2022 |
| Gluconic lactone | Organic acid |  |  | ✔ | Zhou et al., 2022 |
| 4-Vinylphenol dimer | Phenolic compound |  |  | ✔ | Zhou et al., 2022 |
| Erythrose | Sugar |  |  | ✔ | Zhou et al., 2022 |
| alpha-Aminoadipic acid | Amino acid |  |  | ✔ | Evers et al., 2010 |
| Citraconic acid | Organic acid |  |  | ✔ | Juhász et al., 2014; Barnaby et al., 2015; Evers et al., 2010; Sprenger et al., 2018 |
| myo-inositol | Sugar |  |  | ✔ | Juhász et al., 2014; Barnaby et al., 2019; Evers et al., 2010; Sprenger et al., 2018 |
| Melibiose | Sugar |  | ✔ |  | Barnaby et al., 2019 |
| Stachyose | Sugar |  | ✔ |  | Barnaby et al., 2015; Barnaby et al., 2019; Wegener et al., 2017; Sheng et al., 2023 |
| Raffinose | Sugar |  | ✔ |  | Barnaby et al., 2015 |
| Maltose | Sugar |  | ✔ |  | Evers et al., 2010 |
| Pinitol | Sugar |  | ✔ |  | Juhász et al., 2014; Barnaby et al., 2015; Barnaby et al., 2019; Drapal et al., 2017; Sprenger et al., 2016; Toubiana et al., 2020; Yang et al., 2015; Orsák et al., 2021; Zhou et al., 2022; Sheng et al., 2023 |
| Starch | Sugar |  | ✔ |  | Yin et al., 2024 |
| α-kutoglutarate | Organic acid |  | ✔ |  | Toubiana et al., 2020; Yin et al., 2024 |
| Verbascose | Sugar |  | ✔ |  | Juhász et al., 2014 |
| Cellobiose | Sugar |  | ✔ |  | Toubiana et al., 2020 |
| Cellotriose | Sugar |  | ✔ |  | Drapal et al., 2017 |
| Sorbitol | Sugar alcohol |  | ✔ |  | Yang et al., 2015; Barnaby et al., 2015; Sprenger et al., 2016; Drapal et al., 2017; Barnaby et al., 2019; Zhou et al., 2022 |
| Sucrose | Sugar |  | ✔ | ✔ | El-Far et al., 2019 |
| methyl octanoate | Fatty acid |  |  | ✔ | El-Far et al., 2019 |
| 3-Hydroxynorvaline | Amino acid |  |  | ✔ | El-Far et al., 2019; Haas et al., 2020 |
| 10-Hydroxydecanoic acid | Fatty acid |  |  | ✔ | Juhász et al., 2014; Drapal et al., 2017 |
| Guanine | Nucleobase |  |  | ✔ | Drapal et al., 2017; Sprenger et al., 2018 |
| Uracil | Nucleobase |  |  | ✔ | Drapal et al., 2017 |
| Tyrosine | Amino acid |  | ✔ | ✔ | Juhász et al., 2014; Yang et al., 2015; Barnaby et al., 2015; Sprenger et al., 2016; Drapal et al., 2017; Wegener et al., 2017; Barnaby et al., 2019; Haas et al., 2020; Demirel et al., 2020; Orsák et al., 2021 |
| Trigonelline | Alkaloid |  | ✔ |  | Barnaby et al., 2019 |
| Galactose | Sugar |  | ✔ |  | Sprenger et al., 2018 |
| β-galactose | Sugar |  | ✔ |  | Sprenger et al., 2018; Haas et al., 2020 |
| Turanose | Sugar |  | ✔ |  | Juhász et al., 2014; Yang et al., 2015; Barnaby et al., 2015; Sprenger et al., 2016; Drapal et al., 2017; Wegener et al., 2017; Barnaby et al., 2019; Demirel et al., 2020; Orsák et al., 2021 |
| Ribose | Sugar |  | ✔ | ✔ | Haas et al., 2020 |
| Spermidine | Polyamine |  |  | ✔ | Juhász et al., 2014; Yang et al., 2015; Barnaby et al., 2015; Demirel et al., 2020 |
| Spermine | Polyamine |  |  | ✔ | Yang et al., 2015; Barnaby et al., 2019; Toubiana et al., 2020 |
| Thermospermine. | Polyamine |  |  | ✔ | Barnaby et al., 2015 |
| Putrescine | Polyamine |  | ✔ | ✔ | Demirel et al., 2020; Yang et al., 2015 |
| Mannose | Sugar |  | ✔ |  | Demirel et al., 2020; Yang et al., 2015 |
| Inositol | Sugar |  | ✔ |  | Juhász et al., 2014; Yang et al., 2015; Barnaby et al., 2015; Sprenger et al., 2016; Barnaby et al., 2019; Demirel et al., 2020; Da Ros et al., 2020; Toubiana et al., 2020 |
| Ribitol | Sugar |  | ✔ |  | Toubiana et al., 2020 |
| Sedoheptulose | Sugar |  | ✔ |  | Toubiana et al., 2020; Yin et al., 2024 |
| Glucose | Sugar |  | ✔ |  | Yin et al., 2024 |
| myo-ino | Sugar |  | ✔ |  | Toubiana et al., 2020; Yin et al., 2024 |
| Galactaric acid | Organic acid |  | ✔ |  | Yang et al., 2015 |
| Galactonic acid | Organic acid |  | ✔ |  | Yang et al., 2015; Barnaby et al., 2015 |
| Arbutin | Glycoside |  | ✔ |  | Yang et al., 2015; Barnaby et al., 2015; Drapal et al., 2017; Barnaby et al., 2019; Demirel et al., 2020; Da Ros et al., 2020; Toubiana et al., 2020; Orsák et al., 2021; Zhou et al., 2022; Yin et al., 2024 |
| Fructose | Sugar |  | ✔ |  | Yang et al., 2015; Barnaby et al., 2015; Barnaby et al., 2019; Da Ros et al., 2020; Toubiana et al., 2020; Yin et al., 2024 |
| Pyridine, 2-hydroxy- | Organic compound |  | ✔ |  | Yang et al., 2015; Barnaby et al., 2015; Sprenger et al., 2016; Drapal et al., 2017; Barnaby et al., 2019; Demirel et al., 2020; Da Ros et al., 2020; Toubiana et al., 2020; Yin et al., 2024 |
| Mannitol | Sugar alcohol |  | ✔ |  | Juhász et al., 2014; Toubiana et al., 2020; Yin et al., 2024 |
| myo-inositol | Organic acid |  | ✔ |  | Juhász et al., 2014; Da Ros et al., 2020 |
| m-inositol | Glycoside |  | ✔ |  | Demirel et al., 2020; Toubiana et al., 2020 |
| Fructose-6-P | Sugar |  | ✔ |  | Yang et al., 2015 |
| Glucose-6-P | Sugar |  | ✔ |  | Barnaby et al., 2019; Demirel et al., 2020; Toubiana et al., 2020 |
| Glycine | Amino acid |  | ✔ |  | Barnaby et al., 2015; Drapal et al., 2017; Toubiana et al., 2020 |
| Guanosine | Nucleoside |  | ✔ |  | Yang et al., 2015; Barnaby et al., 2015; Barnaby et al., 2019 |
| Uridine | Nucleoside |  | ✔ | ✔ | Demirel et al., 2020 |
| Cytidine | Nucleoside |  | ✔ | ✔ | Barnaby et al., 2019 |
| Cytosine | Nucleoside |  |  | ✔ | Yang et al., 2015 |
| Adenosine | Nucleoside |  | ✔ | ✔ | Evers et al., 2010; Yang et al., 2015; Barnaby et al., 2015; Sprenger et al., 2016; Ren et al., 2017; Drapal et al., 2017; Yooyongwech et al., 2017; Barnaby et al., 2019; Haas et al., 2020; Demirel et al., 2020; Da Ros et al., 2020; Li et al., 2020; Kumar et al., 2020; Toubiana et al., 2020; Yoshida et al., 2020; Orsák et al., 2021; Samy, 2021; Dorneles et al., 2021; Pereira et al., 2022; Koundinya et al., 2024; Dorneles et al., 2024; Yin et al., 2024 |
| 3-Phosphoglyceric acid | Organic compound |  | ✔ |  | Yoshida et al., 2020 |
| Glycerate | Organic compound |  | ✔ |  | Drapal et al., 2017 |
| Leucine | Amino acid |  | ✔ | ✔ | Evers et al., 2010; Yang et al., 2015; Barnaby et al., 2015; Ren et al., 2017; Zhou et al., 2022; Li et al., 2022 |
| Alanine | Amino acid |  | ✔ | ✔ | Juhász et al., 2014 |
| Valine | Amino acid |  | ✔ | ✔ | Juhász et al., 2014; Barnaby et al., 2015, 2019; Yang et al., 2015 |
| Asparagine | Amino acid |  | ✔ | ✔ | Yin et al., 2024 |
| α-aminobutyric acid | Amino acid |  | ✔ |  | Da Ros et al., 2020; Orsák et al., 2021 |
| Glutamate | Amino acid |  | ✔ |  | Da Ros et al., 2020 |
| Phosphoenolpyruvate | Organic acid |  | ✔ |  | Toubiana et al., 2020 |
| Aspartate | Amino acid |  | ✔ |  | Evers et al., 2010; Drapal et al., 2017 |
| Gamma-aminobutyric acid | Amino acid |  | ✔ |  | Evers et al., 2010; Sprenger et al., 2016; Drapal et al., 2017; Haas et al., 2020 |
| Shikimate | Organic acid |  | ✔ |  | Zhou et al., 2022 |
| Galactinol | Sugar |  | ✔ |  | Zhou et al., 2022 |
| Putrescine | Polyamine |  | ✔ |  | Zhou et al., 2022 |
| Adipate | Dicarboxylic acid |  | ✔ |  | Zhou et al., 2022 |
| Proline | Amino acid | ✔ | ✔ | ✔ | Zhou et al., 2022 |
| 5-oxo-Proline | Amino acid |  | ✔ |  | Zhou et al., 2022 |
| Malondialdehyde | Organic compound |  |  | ✔ | Zhou et al., 2022 |
| Threonic acid | Organic acid |  | ✔ |  | Zhou et al., 2022 |
| Gluconic acid | Organic acid |  | ✔ |  | Zhou et al., 2022 |
| Glycerol | Sugar |  | ✔ |  | Zhou et al., 2022 |
| Glycerolgalactose | Sugar |  | ✔ |  | Evers et al., 2010 |
| Lysine | Amino acid |  | ✔ |  | Barnaby et al., 2015; Demirel et al., 2020; Da Ros et al., 2020; Toubiana et al., 2020 |
| Methionine | Amino acid |  | ✔ | ✔ | Da Ros et al., 2020; Toubiana et al., 2020; Yin et al., 2024 |
| Quinate | Organic acid |  | ✔ |  | Yang et al., 2015; Barnaby et al., 2019; Toubiana et al., 2020 |
| dehydroascorbate | Organic compound |  | ✔ |  | Toubiana et al., 2020 |
| Phenylalanine | Amino acid |  | ✔ |  | Juhász et al., 2014; Yang et al., 2015; Drapal et al., 2017; Barnaby et al., 2019; Demirel et al., 2020; Da Ros et al., 2020; Toubiana et al., 2020 |
| Homoserine | Amino acid |  | ✔ |  | Demirel et al., 2020 |
| Malonate |  |  | ✔ |  | Yang et al., 2015; Barnaby et al., 2015; Barnaby et al., 2019 |
| Aspartic acid | Amino acid |  | ✔ | ✔ | Juhász et al., 2014; Yang et al., 2015; Barnaby et al., 2015; Drapal et al., 2017; Barnaby et al., 2019; Yin et al., 2024 |
| p-Coumaric acid | Phenolic acid |  | ✔ |  | Drapal et al., 2017 |
| Caffeic acid | Phenolic acid |  | ✔ |  | Drapal et al., 2017 |
| Neochlorogenic acid | Organic acid |  | ✔ |  | Drapal et al., 2017 |
| Chlorogenic acid | Organic acid |  | ✔ | ✔ | Drapal et al., 2017; Zhou et al., 2022 |
| Crytochlorogenic acid | Organic compound |  | ✔ |  | Drapal et al., 2017 |
| DL-dihydrosphingosine | Sphingolipid |  |  | ✔ | Zhou et al., 2022 |
| ribose-5-phosphate | Phosphate sugar |  |  | ✔ | Zhou et al., 2022 |
| Ferulic acid | Phenolic acid |  | ✔ |  | Drapal et al., 2017 |
| Sinapic acid | Phenolic acid |  | ✔ |  | Drapal et al., 2017 |
| Pyruvate | Organic compound |  | ✔ |  | Yang et al., 2015; Barnaby et al., 2015; Sprenger et al., 2016 |
| Palmitic acid | Fatty acid |  | ✔ |  | Juhász et al., 2014 |
| Stearic acid | Fatty acid |  | ✔ |  | Juhász et al., 2014 |
| glucose-6-phosphate |  |  | ✔ |  | Juhász et al., 2014 |
| Citrate |  |  | ✔ |  | Yang et al., 2015; Barnaby et al., 2015; Barnaby et al., 2019; Toubiana et al., 2020 |
| Quinate | Organic compound |  | ✔ |  | Barnaby et al., 2019 |
| Cis-aconitate | Organic acid |  | ✔ |  | Juhász et al., 2014; Barnaby et al., 2019 |
| Oxaloacetate | Organic acid |  | ✔ |  | Yang et al., 2015 |
| 2-oxoglutaric acid | Organic acid |  | ✔ |  | Barnaby et al., 2019 |
| Inositol | Sugar alcohol |  | ✔ |  | Juhász et al., 2014; Demirel et al., 2020 |
| Malate | Organic acid |  | ✔ |  | Yang et al., 2015; Barnaby et al., 2015; Barnaby et al., 2019; Toubiana et al., 2020 |
| Allantoin |  |  | ✔ |  | Demirel et al., 2020; Da Ros et al., 2020; Toubiana et al., 2020 |
| Isoleucine | Amino acid |  | ✔ | ✔ | Juhász et al., 2014; Yang et al., 2015; Barnaby et al., 2019; Demirel et al., 2020; Toubiana et al., 2020; Yin et al., 2024 |
| Fumarate | Organic acid |  | ✔ |  | Yang et al., 2015; Barnaby et al., 2015; Barnaby et al., 2019; Toubiana et al., 2020 |
| Lactate | Organic acid |  | ✔ |  | Toubiana et al., 2020 |
| β -Fructose | Sugar |  | ✔ |  | Toubiana et al., 2020 |
| β - Glucose | Sugar |  | ✔ |  | Toubiana et al., 2020 |
| α - Fructose | Sugar |  | ✔ |  | Toubiana et al., 2020 |
| α – Glucose | Sugar |  | ✔ |  | Toubiana et al., 2020 |
| Succinate | Organic acid |  | ✔ |  | Yang et al., 2015; Barnaby et al., 2015; Barnaby et al., 2019; Demirel et al., 2020 |
| Trihydroxy pentanoate | Organic compound |  | ✔ |  | Demirel et al., 2020 |
| Glutarate | Organic acid |  | ✔ |  | Barnaby et al., 2015 |
| Cis-aconitate | Organic acid |  | ✔ |  | Yang et al., 2015 |
| 2-oxoglutarate | Organic acid |  | ✔ |  | Yang et al., 2015; Barnaby et al., 2015 |
| Glutamic acid/glutamine | Amino acid |  | ✔ | ✔ | Juhász et al., 2014; Yang et al., 2015; Barnaby et al., 2015; Sprenger et al., 2016; Drapal et al., 2017; Toubiana et al., 2020; Yin et al., 2024 |
| Pyroglutamine | Amino acid |  | ✔ |  | Toubiana et al., 2020 |
| ß-alanine | Amino acid |  | ✔ |  | Juhász et al., 2014 |
| Maleate | Organic acid |  | ✔ |  | Yang et al., 2015; Barnaby et al., 2015; Barnaby et al., 2019 |
| Oxalate | Organic acid |  | ✔ |  | Barnaby et al., 2015 |
| Histidine | Amino acid |  | ✔ | ✔ | Barnaby et al., 2015; Da Ros et al., 2020; Orsák et al., 2021; Yin et al., 2024 |
| 4-Hydroxyphenyl-beta-glucopyranoside | Glycoside |  | ✔ |  | Sprenger et al., 2016; Haas et al., 2020 |
| Arabinonic acid | Organic acid |  | ✔ |  | Sprenger et al., 2016 |
| Arabinose | Sugar |  | ✔ |  | Sprenger et al., 2016 |
| Arabitol | Sugar |  | ✔ |  | Sprenger et al., 2016 |
| Ascorbic acid | Vitamin |  | ✔ |  | Sprenger et al., 2016 |
| Benzoic acid | Organic acid |  | ✔ |  | Sprenger et al., 2016 |
| Butanoic acid, 4-amino- | Amino acid |  | ✔ |  | Sprenger et al., 2016 |
| Benzoic acid, 3-hydroxy- | Phenolic acid |  | ✔ |  | Haas et al., 2020 |
| Benzoic acid, 3,4-dihydroxy- | Phenolic acid |  | ✔ |  | Sprenger et al., 2016 |
| Butyric acid, 4-amino- | Amino acid |  | ✔ |  | Sprenger et al., 2016 |
| Caffeic acid, trans- | Phenolic acid |  | ✔ |  | Sprenger et al., 2016 |
| Citric acid | Organic acid |  | ✔ |  | Sprenger et al., 2016 |
| Isocitric acid | Organic acid |  | ✔ |  | Sprenger et al., 2016; Drapal et al., 2017 |
| Dehydroascorbic acid dimer | Vitamin |  | ✔ |  | Drapal et al., 2017 |
| Dopamine | Organic acid |  | ✔ |  | Sprenger et al., 2016 |
| Erythronic acid | Sugar |  | ✔ |  | Sprenger et al., 2016 |
| Ethanolamine | Organic acid |  | ✔ |  | Sprenger et al., 2016; Haas et al., 2020 |
| Ferulic acid, trans- | Phenolic acid |  | ✔ |  | Sprenger et al., 2016 |
| Fructose-6-phosphate | Sugar |  | ✔ |  | Sprenger et al., 2016 |
| Tyramine | Amino acid |  | ✔ | ✔ | Sprenger et al., 2016 |
| Fumaric acid | Organic acid |  | ✔ |  | Sprenger et al., 2018; Da Ros et al., 2020; Yin et al., 2024 |
| Glucaric acid-1,4-lactone | Organic acid |  | ✔ |  | Juhász et al. 2014; Sprenger et al. 2016; Drapal et al. 2017; Sprenger et al. 2018 |
| Maleic acid | Organic acid |  | ✔ |  | Sprenger et al. 2018 |
| Dopamine | Sugar |  | ✔ |  | Drapal et al. 2017 |
| Glutaric acid | Organic acid |  | ✔ |  | Sprenger et al. 2018 |
| isocitric acid | Organic acid |  | ✔ |  | Juhász et al. 2014 |
| Erythronic acid | Organic acid |  | ✔ |  | Sprenger et al. 2018 |
| Galactaric acid | Organic acid |  | ✔ |  | Juhász et al. 2014; Sprenger et al. 2016; Haas et al. 2020 |
| Galactinol | Organic acid |  | ✔ |  | Juhász et al. 2014; Sprenger et al. 2016 |
| Galactonic acid | Organic acid |  | ✔ |  | Sprenger et al. 2016 |
| Gluconic acid | Organic acid |  | ✔ |  | Sprenger et al. 2016 |
| Glucose-6-phosphate | Sugar |  | ✔ |  | Sprenger et al. 2016 |
| Glutaric acid, 2-oxo- | Organic acid |  | ✔ |  | Sprenger et al. 2016 |
| Glyceric acid | Organic acid |  | ✔ |  | Juhász et al. 2014; Sprenger et al. 2016; Drapal et al. 2017; Sprenger et al. 2018; Haas et al. 2020 |
| Glucopyranose | Sugar |  | ✔ |  | Haas et al. 2020 |
| Glyceric acid-3-phosphate | Organic acid |  | ✔ |  | Sprenger et al. 2016 |
| Glycerate | Organic acid |  | ✔ |  | Barnaby et al. 2019 |
| Glycolic acid | Organic acid |  | ✔ |  | Sprenger et al. 2016 |
| Hexadecanoic acid | Fatty acid |  | ✔ |  | Sprenger et al. 2016 |
| Hexanoic acid, 2-ethyl- | Fatty acid |  | ✔ |  | Sprenger et al. 2016 |
| Inositol-1-phosphate | Phosphate sugar |  | ✔ |  | Sprenger et al. 2016 |
| Inositol, myo- | Sugar |  | ✔ |  | Juhász et al. 2014; Sprenger et al. 2016 |
| Octopamine | Amino acid |  | ✔ |  | Sprenger et al. 2016 |
| Phosphoric acid | Inorganic acid |  | ✔ |  | Sprenger et al. 2016 |
| Phosphoric acid monomethyl ester | Phosphate compound |  | ✔ |  | Sprenger et al. 2016 |
| Putrescine | Polyamine |  | ✔ |  | Haas et al. 2020 |
| Pyridine, 2-hydroxy- | Organic compound |  | ✔ |  | Sprenger et al. 2016 |
| Pyroglutamic acid (Glutamine, Glutamic acid) | Amino acid |  | ✔ |  | Sprenger et al. 2016 |
| Quinic acid | Organic acid |  | ✔ | ✔ | Juhász et al. 2014; Sprenger et al. 2016; Haas et al. 2020; Da Ros et al. 2020 |
| Quinic acid, 3-caffeoyl-, cis- | Phenolic compound |  | ✔ |  | Sprenger et al. 2016; Drapal et al. 2017; Haas et al. 2020; Zhou et al. 2022 |
| Quinic acid, 3-caffeoyl-, trans- | Phenolic compound |  | ✔ |  | Sprenger et al. 2016 |
| Quinic acid, 4-caffeoyl-, cis- | Phenolic compound |  | ✔ |  | Sprenger et al. 2016 |
| Quinic acid, 4-caffeoyl-, trans- | Phenolic compound |  | ✔ |  | Sprenger et al. 2016; Haas et al. 2020 |
| Quinic acid, 5-caffeoyl-, trans- | Phenolic compound |  | ✔ |  | Sprenger et al. 2016 |
| Ribitol | Sugar |  | ✔ |  | Sprenger et al. 2016 |
| Ribonic acid | Organic acid |  | ✔ |  | Sprenger et al. 2016; Haas et al. 2020 |
| Saccharic acid | Organic acid |  | ✔ |  | Sprenger et al. 2016; Sprenger et al. 2018; Haas et al. 2020 |
| Umbelliferone | Phenolic acid |  | ✔ |  | Sprenger et al. 2016 |
| Rutin | Flavonoid |  | ✔ |  | Sprenger et al. 2016; Sprenger et al. 2018; Haas et al. 2020 |
| Naringin | Flavonoid |  | ✔ |  | Drapal et al. 2017 |
| Phytoene | Carotenoid |  | ✔ |  | Drapal et al. 2017 |
| b-carotene | Carotenoid |  | ✔ |  | Drapal et al. 2017 |
| Anheraxanthin | Carotenoid |  | ✔ |  | Drapal et al. 2017 |
| Violaxanthin | Carotenoid |  | ✔ |  | Drapal et al. 2017 |
| Neoxanthin | Carotenoid |  | ✔ |  | Drapal et al. 2017 |
| Lutein | Carotenoid |  | ✔ |  | Drapal et al. 2017 |
| Pheophytin |  |  | ✔ |  | Drapal et al. 2017 |
| Tryptophan | Amino acid |  | ✔ | ✔ | Drapal et al. 2017 |
| Salicylic acid-glucopyranoside | Organic acid |  | ✔ |  | Drapal et al. 2017 |
| Serine | Amino acid |  | ✔ | ✔ | Drapal et al. 2017 |
| Shikimic acid | Organic acid |  | ✔ |  | Juhász et al. 2014; Drapal et al. 2017; Toubiana et al. 2020; Yin et al. 2024 |
| Itaconic acid | Organic acid |  | ✔ |  | Sprenger et al. 2016 |
| Mesaconic acid | Organic acid |  | ✔ |  | Juhász et al. 2014; Yang et al. 2015; Barnaby et al. 2019; Sprenger et al. 2016; Drapal et al. 2017; Haas et al. 2020; Demirel et al. 2020; Da Ros et al. 2020; Yin et al. 2024 |
| Succinic acid | Organic acid |  | ✔ |  | Sprenger et al. 2016; Drapal et al. 2017; Haas et al. 2020 |
| Threonic acid | Organic acid |  | ✔ |  | Drapal et al. 2017 |
| Threonic acid-1,4-lactone | Organic acid |  | ✔ |  | Drapal et al. 2017 |
| Threonine | Amino acid |  | ✔ | ✔ | Sprenger et al. 2016; Drapal et al. 2017; Sprenger et al. 2018 |
| Tyramine | Amino acid |  | ✔ | ✔ | Sprenger et al. 2016; Haas et al. 2020 |
| Lactic acid | Organic acid |  | ✔ |  | Sprenger et al. 2016 |
| Malic acid | Organic acid |  | ✔ |  | Juhász et al. 2014; Orsák et al. 2021; Yang et al. 2015; Barnaby et al. 2015; Da Ros et al. 2020; Toubiana et al. 2020; Yin et al. 2024; Sprenger et al. 2016; Drapal et al. 2017; Yin et al. 2024 |
| Malic acid, 2-methyl- | Organic acid |  | ✔ |  | Sprenger et al. 2016; Sprenger et al. 2018; Da Ros et al. 2020; Yin et al. 2024 |
| Noradrenaline | Catecholamine |  | ✔ |  | Sprenger et al. 2016 |
| Octadecanoic acid | Fatty acid |  | ✔ |  | Sprenger et al. 2016; Drapal et al. 2017; Haas et al. 2020 |

✔ = indicate the presence of the metabolite in a particular tuber crop.


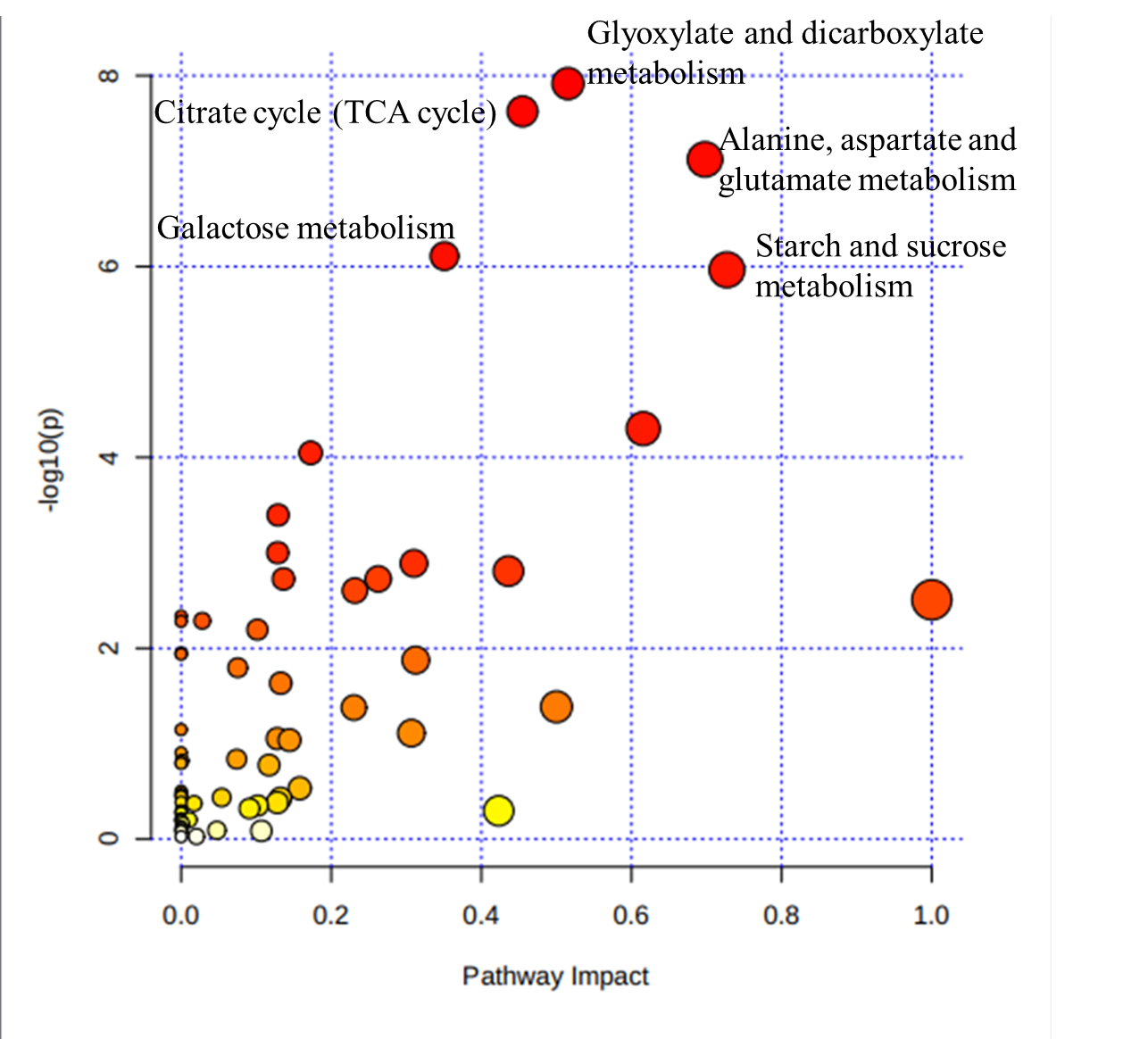
Figure S1: Pathway analysis using all identified metabolites in major tuber crop showing metabolic pathways represented as nodes. The graph presents a view of all the matched pathways arranged by p-values on the y-axis, and the pathway impact values on the x-axis. The node colour (beige to red) is based on the node's p-value, and the node radius is defined by the pathway impact values. A pathway impact value > 0.1 and p < 0.05 was considered a target.
